# Bayesian fine-mapping pinpoints candidate genes and pleiotropic loci of production traits from a chicken backcrossing scheme

**DOI:** 10.1186/s12864-026-13069-z

**Published:** 2026-06-16

**Authors:** Chi Mei Sun, Johannes Geibel, Henner Simianer, Björn Andersson, David Cavero, Rudolf Preisinger, Steffen Weigend, Christian Reimer

**Affiliations:** 1 Friedrich-Loeffler-Institut – Institute of Farm Animal Genetics, Höltystraße 10, Neustadt, 31535 Germany; 2 Center for Integrated Breeding Research, University of Göttingen, Albrecht-Thaer-Weg 3, Göttingen, 37075 Germany; 3 Lohmann Breeders GmbH, Am Seedeich 9-11, Cuxhaven, 27472 Germany; 4 H&N International GmbH, Am Seedeich 9-11, Cuxhaven, 27472 Germany; 5 EW GROUP GmbH, Hogenbögen 1, Visbek, 49429 Germany; 6 Animal Breeding and Genetics Group, University of Göttingen, Albrecht-Thaer-Weg 3, Göttingen, 37075 Germany

**Keywords:** GWAS, Bayesian fine-mapping, Egg production, Egg weight, Body weight, Chicken, Backcross

## Abstract

**Background:**

Understanding the genetic architecture of economically important traits in poultry is critical for improving breeding strategies. In this study, we investigated a backcrossing scheme between a White Layer line and Araucana chickens. Genome-wide association studies (GWAS) were performed using array-genotyped and imputed data. We also explored the use of correlated traits as covariates, which helps to distinguish between pleiotropic and trait-specific associations. We applied two Bayesian fine-mapping methods to refine GWAS-identified QTLs and pinpoint candidate variants and genes associated with egg number (EN, from 20 to 71 weeks), egg weight (EW, from 30 to 70 weeks), and body weight (BW, at 32 weeks): the forward selection approach and functional annotation enrichment implemented in BFMAP, and the shotgun stochastic search algorithm implemented in FINEMAP.

**Results:**

GWAS identified multiple significant loci associated with BW and EW, with high heritability estimated for these traits. EN showed a more polygenic architecture with lower heritability across most periods. Including correlated traits as covariates in GWAS revealed pleiotropic loci, particularly on chromosomes 1 and 4, that influenced both BW and EW, as well as loci specific to individual traits. Both fine-mapping methods successfully pinpointed candidate genes such as *NCAPG*, *LCORL*, and *IGF2BP1*, which are well known for their roles in growth and body size across species. Several novel candidate genes were also highlighted for EN. Notably, some fine-mapped results reflected patterns consistent with the covariate-adjusted GWAS results.

**Conclusions:**

This study demonstrates the power of combining GWAS with imputation and fine-mapping methods in chickens to uncover the genetic basis of economically important traits. Furthermore, incorporating correlated traits as covariates in GWAS provided valuable insights, enabling the distinction between pleiotropic and trait-specific loci. Together, these approaches refine GWAS signals and deepened our understanding of the genetic architecture underlying complex traits.

**Supplementary Information:**

The online version contains supplementary material available at 10.1186/s12864-026-13069-z.

## Background

The introduction of single nucleotide polymorphism (SNP) arrays has significantly advanced the genetic analysis of economically important traits in chickens, offering unprecedented insights into their complex genetic architecture. In genome-wide association studies (GWAS), SNP arrays serve as powerful tools for identifying novel loci associated with phenotypic variation across different chicken lines, including egg production and quality [1, 2], growth [3, 4], meat quality [5], and disease resistance [6]. These advancements have identified quantitative trait loci (QTLs) and candidate genes linked to key traits of laying chickens, such as egg production and quality, and body weight, that can drive genetic improvements in poultry breeding [7–13].

Despite these achievements, challenges remain. Although SNP arrays are cost-effective and widely used, their medium density limits the resolution for fine-mapping causal variants. One way to overcome this limitation is through imputation, where lower density SNP arrays are imputed to whole-genome sequence (WGS) level using reference panels of related sequenced animals. In chickens, several studies have incorporated imputation in GWAS to enhance detection power [14–16]. This approach provides denser genomic information and, according to a simulation study, can substantially boost GWAS power, enabling commercial SNP arrays to perform close to an ideal “complete” chip [17]. However, empirical studies suggest that while WGS-based GWAS enables the discovery of novel variants missed by array GWAS [18, 19], denser data often reinforce signals from known QTLs and may be penalized by a more stringent significance threshold [20]. Despite these differing perspectives on the benefits of imputation, there is broad consensus that increasing sample size remains one of the most effective strategies for improving detection power in GWAS [17, 20–22]. In practice, imputation is best viewed as a complementary tool to enhance marker density and fine-mapping resolution, rather than a guaranteed means of detecting novel loci.

Another fundamental limitation for GWAS resolution is that a detected QTL contains numerous genes rather than pinpointing candidate genes with precision, due to the extent of linkage disequilibrium (LD) and generally long haplotypes in structured populations. However, studies have demonstrated that repeated intercrossing of outbred lines can enhance QTL mapping resolution for growth traits such as body weight [23–26], which suggests that introducing recombination events help reduce QTL intervals. GWAS methodologies can also be adapted to improve resolution and interpretation. Including covariates in GWAS is a common approach to control for confounding factors such as population structure and environmental influences that could otherwise bias the identification of the true genetic associations. Covariates such as principal components, age, sex, batch effect, lead SNPs, and correlated traits were generally considered to avoid stratification [16, 26–29]. Beyond just addressing confounding, multi-trait GWAS approaches have been increasingly explored, allowing to detect pleiotropic loci that may not reach the significance thresholds in single-trait GWAS analyses [20, 30, 31].

While traditional GWAS is effective for detecting loci with strong effects, it faces limitations in detection of polygenic traits where many SNPs contribute small individual effects, or when LD complicates the identification of causal variants. Bayesian methods offer a solution by incorporating prior probabilities and modelling sparse SNP effects, thereby refining the large QTLs identified through GWAS. Several Bayesian fine-mapping methods have been applied to refine GWAS-identified QTLs. Approaches that integrate forward selection and functional enrichment, such as BFMAP, have successfully revealed candidate genes for important traits in cattle [32–34]. Other methods, such as FINEMAP [35], focus on shotgun stochastic search algorithm [36].

The objective of this study was to dissect the genetic architecture underlying production traits through integration of different GWAS methodologies and Bayesian fine-mapping tool, focused on a backcrossing and intercrossing scheme between Araucana chickens and high-performing White Layer (WL) hens.

## Materials and methods

### Animals and phenotypes

**Fig. 1:**
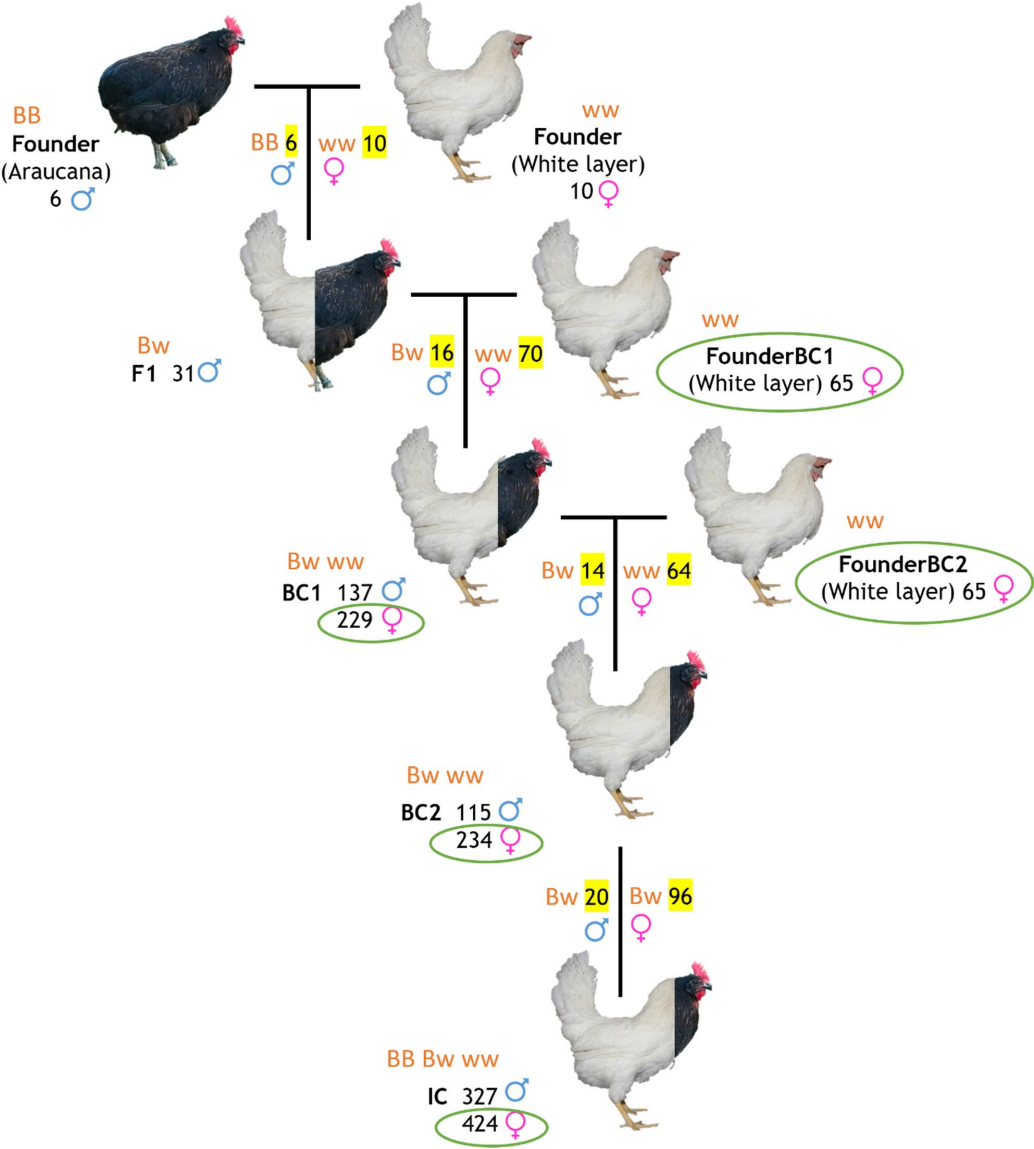
The back-crossing scheme between Araucana chickens and commercial White Layers (WL). The black lines represent the pedigree relationships between individuals across generations, information next to the black lines indicates the parent’s characteristics. BB, Bw and ww refer to the genotypes of the blue eggshell insertion where B refers to the color allele. Integers indicating the number of animals selected as parents (highlighted) or genotyped (not highlighted) in the corresponding generation. Phenotypes were measured on females from five generations (in green circles)

The animals used in this study are owned by the breeding company Lohmann Breeders GmbH. This study focused on a backcrossing scheme between distinct breeds, developed as part of the EU Horizon 2020 project Innovative Management of Animal Genetic Resources (IMAGE; https://www.imageh2020.eu/). This backcrossing scheme was designed to introgress blue eggshell colour from Araucana chickens into a high-performing White Layer line. The blue eggshell colour is determined by the autosomal inheritance of the *SLCO1B3* gene (1:65167287–65185448) [37], “BB”, “Bw” and “ww” refer to the genotypes of the blue eggshell insertion where “B” refers to the color allele. Six Araucana males were crossed with ten high-performing White Layer (WL) females from a White Leghorn line to produce F1, followed by two back-crossing generations with WL hens to produce BC1 and BC2, and intercrossing within BC2 to produce the IC generation (Fig. 1). Only males of F1 and BC1 were selected to mate with white layer hens for producing BC1 and BC2. Parents from F1, BC1 and BC2 were selected as the carrier of the color allele (Bw).

Phenotypic traits were recorded in hens from BC1, BC2, IC and the WL founders of the BC cohorts (FounderBC1, FounderBC2), data is available in Supplementary Data 1 and in Supplementary Figs. 4. Only eggs with intact shell were selected for measuring egg quality traits. Egg number (EN) was recorded in thirteen 28-day periods, starting from 20 weeks (w) of age as the first period (EN1; 20w to 24w), measured as the total number of eggs during the corresponding period. There was no record for EN9 where the selection of individuals took place. Egg weight (EW) was measured at 30, 40, 50 and 70 weeks of age as the average weight of a 3-day clutch, although “FounderBC1” and “BC2” did not have data for EW50. Body weight (BW) was a onetime measurement at 32 weeks of age (Table 1).

**Table 1:** Descriptive statistics for phenotypes in all generations

Trait	*N*	Weeks	Mean	SD	CV(%)	Min(%)	Max(%)
EN1	874	20–23	11.86	9.02	76.1	0(19.6%)	28(1.8%)
EN2	874	24–27	24.12	4.9	20.3	0(0.8%)	28(23.5%)
EN3	874	28–31	25.61	4.4	17.2	0(1.1%)	28(35.4%)
EN4	874	32–35	25.68	4.8	18.7	0(1.6%)	28(39.7%)
EN5	874	36–39	25.38	5.31	20.9	0(2.5%)	28(35.7%)
EN6	874	40–43	25.51	5.13	20.1	0(2.2%)	28(35.9%)
EN7	874	44–47	25.32	5.26	20.8	0(2.4%)	28(33.5%)
EN8	874	48–51	24.62	5.8	23.6	0(2.9%)	28(28.5%)
EN10	854	56–59	23.95	5.91	24.7	0(1.6%)	28(20.0%)
EN11	854	60–63	23.56	6.49	27.6	0(2.9%)	28(20.5%)
EN12	854	64–67	22.75	7.17	31.5	0(4.6%)	28(15.5%)
EN13	853	68–71	21.69	7.59	35.0	0(5.4%)	28(12.2%)
BW32	864	32	1786.2	207.53	11.6	1202(0.1%)	2582(0.1%)
EW30	854	30	57.66	4.54	7.9	45(0.1%)	73(0.1%)
EW40	831	40	61.39	4.51	7.4	50(0.1%)	79(0.1%)
EW50	524	50	62.1	4.72	7.6	51(0.2%)	79(0.2%)
EW70	746	70	64.02	5.03	7.9	52(0.1%)	80(0.1%)

There was no record for EN9 where the selection of individuals took place

*N* Number of records, *SD* Standard deviation, *CV(%)* Coefficient of variance in percentage, Min(%)/Max(%) – minimum/maximum value (proportion of individuals with that value)

### Genotyping and array-based GWAS

Araucana founders (6 males) were sequenced and WL founders (10 females) were genotyped using Affymetrix Axiom 600k array [38]. Remaining animals were genotyped for 52k SNPs using a custom Affymetrix array (ThermoFisher, Santa Clara, CA, USA). Overlapping SNPs were combined and genotypes were mapped to the chicken reference genome GRCg6a. Genotype data were filtered using PLINK 1.9 [39], by removing samples and SNPs with missing call rate greater than 15%. The iltered genotype data contained 46,605 SNPs and 1,639 individuals with 1,024 females and 615 males.

Genome-wide association studies (GWAS) for EN, EW and BW measured throughout different periods were performed based on the filtered genotype data and different sample groups, including all the animals with non-missing phenotypic records (WL founders for BC1 and BC2, BC1, BC2, IC) denoted as group “all”, and group “offspring” included BC1, BC2 and IC, and by each generation. BW32 was included as a quantitative covariate in the analyses of EW traits, and the corresponding EW measurement was included as a covariate in the analyses of BW32.

Array-based GWAS (array GWAS) was conducted for each trait including the generation as a fixed effect, using single-marker regression with polygenic correction by GCTA --mlma [40, 41], and the genetic relationship matrix (GRM) for GWAS was computed between all the animals (1,639) using array genotypes, by GCTA --make-grm-bin [40, 42] (GCTA version v1.94.1). Additional minor allele frequency (MAF) filtration was applied on each GWAS to remove SNPs with MAF lower than 0.01. The filtered genotyped data was fitted to the mixed linear model:

$$y=X\beta\:+g+e$$

where $\:y$ is a vector of phenotypes of animals; $\beta$ is a vector of fixed effects; $X$ is the design matrix for fixed effects; $g$ is a vector of random genetic effects that captures the total genetic variation with $g\:\sim\:N(0,\:G{\sigma}_{g}^{2})$, where $G$ is the computed GRM; $e$ is a vector of residuals with $e\:\sim\:N(0,\:I{\sigma}_{e}^{2})$. We applied a strict genome-wide Bonferroni-corrected threshold, calculated as *P* < 0.05 divided by the total number of tested SNPs for each analysis [43].

### Imputation and accuracy

The filtered genotype data were phased and imputed using Beagle 5.4 [44, 45] with a reference panel of nine Araucana (of which six were the founders for the IMAGE backcross) and 161 White Leghorn samples and commercial White Layer lines, retrieved from a global reference set of around 5k chickens, prepared by the Chicken Genomic Diversity Consortium [46]. Since the Genotype data was mapped to GRCg6a, the reference sequence was lifted from GRCg7b to GRCg6a by bcftools-1.20 [47] using a custom chain file prepared according to [48]. The snakemake pipeline for the liftover is available on 10.5281/zenodo.15343279.

To visualise the relationship between the target and reference populations, we performed principal component analysis (PCA) on the merged target genotype and reference sequence data used for imputation. A set of 922,693 LD-pruned SNPs was generated by PLINK 1.9 with the --indep-pairwise 100 50 0.3 option [39], and PCA was conducted on the maximum number of principal components. The resulting plots are presented in Supplementary Fig. 3.

Imputation produced approximately 11 million biallelic variants. Chromosomes (chr) 16 and Z were not available in the reference data and were therefore excluded from the imputed results. Imputation accuracy was accessed using five-fold cross-validation with two repetitions, by masking a subset of variants from the array-genotyped data in each fold and comparing the masked genotypes with the imputed ones. The mean concordance was 0.95, and the mean Pearson’s correlation was 0.92. The average Imputation Quality Score (IQS), which adjusts for the minor allele frequency [49], was 0.88, while the average predicted imputation quality (dosage r-square; DR2) was 0.77. Accuracy was also evaluated across MAF bins (0, 0.01, 0.05, 0.1, 0.3, 0.5). Mean concordance decreased slightly with increasing MAF, ranging from 0.94 to 0.99. In contrast, mean Pearson’s correlation, IQS, and DR2 increased with MAF, ranging from 0.77 to 0.94, 0.44 to 0.91, and 0.26 to 0.80, respectively. A detailed summary is provided in Result 1 of Supplementary Data 2. For subsequent analyses, we retained 4,868,808 (44.4%) ariants with good predicted imputation quality (DR2 ≥ 0.8).

### Imputation-based GWAS

GWAS based on the 4,868,808 imputed variants (imputed GWAS) was conducted for each trait, including generation as a fixed effect. A dense GRM was computed using the same method as in the array GWAS, based on all 1,639 animals with imputed genotypes. GWAS was performed using the same mixed linear model (GCTA --mlma) for each trait and sample group with non-missing phenotypic records. Additional minor allele frequency (MAF) filtration was applied on both analyses to remove SNPs with MAF lower than 0.01. The genome-wide Bonferroni-corrected threshold became stricter (0.05 divided by the total number of tested SNPs for each analysis) as the number of SNPs significantly increased. Additionally, BW32 was included as a quantitative covariate in both array and imputed GWAS of EW traits, and vice versa.

The genomic inflation factors were computed based on *p*-values from LD-pruned SNPs (PLINK 1.9 --indep-pairwise 100 50 0.3) in both array and imputed GWAS summary statistics, for each trait and sample group combination. The mean inflation factors by generation ranged from 0.93 to 1.1, detailed summary was documented in Result 2 of Supplementary Data 2.

### Multi-trait metaGWAS

A multi-trait metaGWAS was conducted on all EN measurements (EN1 – EN13, excluding EN9 due to missing data) using the summary statistics from imputed GWAS. The formula $Multi\:trait\:{X}^{2}={t}_{i}^{{\prime}}{V}^{-1}{t}_{i}$ suggested by Bolormaa et al. [30], where each SNP was assigned a t-value calculated as the allele effect divided by the standard error obtained from the imputed-based GWAS $(t=\frac{b}{se})$, while ${t}_{i}$ is an $n*1$ vector of t-values of ${SNP}_{i}$ for the $n$ traits, ${t}_{i}^{{\prime}}$ is a transpose of the ${t}_{i}\:\left(1*n\right)$, ${V}^{-1}$ is an inverse of the $\:n*n$ correlation matrix between all the traits calculated based on the signed t-values of the SNPs. The genome-wide Bonferroni-corrected threshold was applied to identify significant variants.

### Phenotypic correlations and genetic parameters

Phenotypic correlations r_p_ between traits after adjusted by generation means, were calculated as Pearson’s correlations by R (version 4.3.2) [50] using function *cor* with *pairwise.complete.obs*, the corresponding *p*-values for the phenotypic correlations were calculated using *cor.mtest* from R package “corrplot” (version 0.92) [51].

Genetic correlations r_g_ between traits were estimated from variance captured by the imputed variants including generation as a fixed effect, with the same model as described in the Array GWAS section, using restricted maximum likelihood analysis. We used Bivariate GREML analysis implemented in GCTA [40, 52, 53] that utilised the GRM computed between all animals (1639) using the imputed genotypes with MAF filtration of 0.01 [42]. GCTA only considered individuals with non-missing phenotypes, therefore the number of samples included in the analysis varied depending on the corresponding phenotypic records.

Genome-wide heritability (*h*^*2*^) for each trait was estimated using BFMAP --varcomp based on imputed variants including generation as a fixed effect. This analysis utilised a GRM computed between all animals across generations according to Yang’s formula [42], with MAF filtration of 0.01. BFMAP employs an eigendecomposition approach to estimate *h*^*2*^ as the ratio ${\sigma}_{g}^{2}/{(\sigma}_{g}^{2}\:+{\sigma}_{e}^{2})$, which is less prone to convergence issues compared to maximum likelihood estimation in our data. However, as a limitation, BFMAP does not provide standard errors or confidence intervals for the resulting h² estimates.

### Fine-mapping analysis

We defined the QTL for fine-mapping based on the distance between the first and the last significant SNP that passed the Bonferroni threshold identified by imputed GWAS in group “all”, with a 1 Mb extension both upstream and downstream, resulted in QTL regions ranging from 2 Mb to 5 Mb. In total, there were 19 QTLs from 17 traits.

We used two Bayesian fine-mapping methods: BFMAP-FS (Version 0.91) [32], which implements forward selection method, and FINEMAP-sss (Version 1.4.2) [35], which employs a shotgun stochastic search algorithm [36]. To inspect the sensitivity to window size of these two methods in our data, we performed analyses using three QTL regions definitions for the traits representing the beginning and ending periods of the phenotypes (BW32, EW30, EW70, EN1, EN13), to compare lead SNP variation: the original window, and windows extended by 1 Mb and 2 Mb upstream and downstream of the original boundaries to ensure inclusion of the lead SNP from the original QTL. Lead SNP was determined as the one with the highest log_10_(Bayes Factor; BF) and posterior probability. The summary table was documented in Result 3 of Supplementary Data 2.

### FINEMAP

For FINEMAP, summary statistics from the imputed GWAS of the “all” group were used as input. SNP correlation matrices for each QTL region were computed using LDstore (Version 2.0) [54] based on the same imputed variants. FINEMAP was run with default settings, assuming 1 to 5 causal SNPs per region. The prior probability of causality for any given SNP was set to $1/m$, where $\:m$ is the number of variants in the region.

FINEMAP employs a shotgun stochastic search algorithm [36] to explore the model space and generate credible configuration sets (CS), which were the smallest sets of configurations whose cumulative posterior probability reached 0.95. For each CS, FINEMAP reports: (1) a posterior probability for the tested number of causal SNP(s), (2) the log_10_(BF) quantifying evidence for an additional causal signal, and (3) the mean absolute correlation between the lead SNP(s) and other SNPs within the CS. Posterior probabilities across all explored CS sum to 1.

For each SNP, FINEMAP calculates a marginal posterior inclusion probability (PIP) across all CS, as well as log_10_(BF) showing the evidence of SNP causality (following Benner et al. [35], a log_10_(BF) > 2 was considered as evident). The count of variants with PIP > 0 (FINEMAP_var) in addition to log_10_(BF) ≥ 2 (FINEMAP_var_evi) were documented in Result 4 of Supplementary Data 2, whereas only those considered as evident were recorded in Supplementary Data 5. The summed PIP at gene level were also generated based on PIP of SNPs, with 1 kb extension both upstream and downstream of the gene [55]. All genes with summed PIP over 0.01 were documented in Supplementary Data 3.

### BFMAP

The phenotypes and imputed genotypes of individuals with complete records, as well as the genome-wide *h*^*2*^ estimated for each trait described in the previous section, were used as input for the following BFMAP analyses. Regional *h*^*2*^ for each QTL was estimated as the proportion of variance explained by SNPs within the QTL region, using a regional GRM computed from QTL SNPs across all animals with MAF filtration of 0.01 according to Yang’s formula [42], and including generation as a covariate. The Bayesian model developed for fine-mapping is:

$$y=Xb+Za+g+e$$

$$b\:\sim\:N(0,\:\varphi\:{\sigma}_{e}^{2}I)$$

$$a\:\sim\:N(0,\:\gamma{\sigma}_{e}^{2}I)$$

$$g\:\sim\:N(0,\:\eta\:{\sigma}_{e}^{2}G)$$

$$e\:\sim\:N(0,\:{\sigma}_{e}^{2}R)$$

$$P\left({\sigma}_{e}^{2}\right)\propto\:1/{\sigma}_{e}^{2}$$

where $y$ is a vector of a phenotype from n animals; $b$ is a vector of covariate effects (generation) and $X$ is the corresponding design matrix; $a$ is a vector of variant effects and $Z$ is the corresponding genotype coding matrix; $g$ is a vector of polygenic effect considering the population structure and $G$ is the corresponding GRM; $e$ is the residual and $R$ is a diagonal matrix used to model differential reliability among animals $({R}_{ii}=\frac{1}{{r}^{2}}-1)$. The ${\sigma}_{e}^{2}$ was assigned a non-informative Jeffrey’s prior. $\varphi$ and $\gamma$ were set to 10^8^, and $\eta$ was set to ${h}^{2}/(1-{h}^{2})$.

Probability of the null model P(D|M_0_) and the probability of the model with variant inclusion given the data P(D|M) can be computed to obtain the scaled Bayes factor and the corresponding p-value for a SNP. BFMAP approximates flat priors for SNP effects [32].

For fine-mapping, BFMAP first performs an iterative forward selection process to identify independent signals, by sequentially adding lead SNPs that were evaluated conditional on all previously selected lead SNPs to the model. The process continues until no variants meet the software’s predefined threshold (2logBF + 1 < 2logm_eff_), with each selected lead SNP represents the proxy of an independent signal. Next, each signal is expanded to include variants in high LD (default r^2^ > 0.3) with the signal proxy. The final lead SNP for each signal is then re-evaluated and determined through repositioning within each signal set. Finally, a posterior conditional inclusion probability (PCIP_*i*_) is assigned to each variant within a signal conditioning on the lead SNPs from all other signals while excluding the lead SNP of that signal. PCIP values are then sorted in descending order, starting from top, a list of variants with the cumulative sum of their PCIP over 95% consists a CS for each signal [32]. The summed PCIP for a gene can also be generated based on the PCIP_*i*_, variants with 1 kb extension both upstream and downstream of the gene were also included [55]. All genes with summed PCIP over 0.01 were documented in Supplementary Data 3. Afterwards we combined all signal-specific CS into a final CS for a QTL.

### Functional enrichment of BFMAP fine-mapping results

Functional enrichment is a special downstream analysis feature from BFMAP on its produced fine-mapping results, that can be conducted using the R package “gemrich” (version 0.1.3) [55]. This package is designed to integrate functional annotations with the forward selection approach of BFMAP. Variant functional annotations were obtained with the Ensembl Variant Effect Predictor (VEP, version 112) [56], applied to variants included in the CS determined by BFMAP. The categories analyzed included Consequences, Impact, and Biotype. The number of variants analyzed in each category could vary slightly dependent on the availability of annotation.

Signal filtration was applied using a p-value threshold that targeted the lead variant of the signal, thereby a signal was either retained or filtered out as a whole. We applied a threshold of *P* < 5 × 10⁻⁵ as recommended by the author. All variants within retained signals were included in the following analyses.

The estimated probability of causal variants being in a category $\left(\hat{{P}_{C}}\right)$ was generated using maximum likelihood based on the produced PCIP_*i*_. The enrichment factor for each category $\left({f}_{C}\right)$ quantified how enriched the candidate causal variants in that category compared to its background frequency $\left({q}_{C}\right)$: ${f}_{C}=\:\frac{\hat{{P}_{C}}}{{q}_{C}}$. To ensure robust enrichment estimates, we excluded runs where fewer than 10 variants remained after signal filtration, or where fewer than two category types were present; these cases were flagged as “Failed” in the “mle_status” column, with the reason noted in the “reason” column. In addition, categories representing less than 1% of variants were grouped into a single “remaining” category. While we observed a high number of failures due to small remained datasets for each trait, we then combined CS from related traits with similar QTL regions — ENs (EN1 – EN13; *QTL-chr2*); BWEWs (BW32, EW30, EW40, EW50, EW70; *QTL-chr4*) — to estimate the enrichment factors together with the combined datasets. Parameters for enrichment estimation were documented in Supplementary Data 4.

For a total of *n* variants remaining, we then renormalized PCIP_*i*_ for each variant *i* to account for the corresponding category enrichment $\left({f}_{C}\right)$, weighted by the total PCIP of $n$ variants:

$${renormedPCIP}_{i}=\frac{{PCIP}_{i}\times{f}_{C}}{\sum\:_{i}^{n}({PCIP}_{i}\times{f}_{C})}\times\:\sum\limits_{i}^{n}{PCIP}_{i}$$

Variants within filtered signals, along with their PCIP_*i*_ and renormedPCIP_*i*_, are provided in Supplementary Data 5. When computing the summed PCIP for a gene based on the filtered variants’ renormedPCIP_*i*_, we included variants within the gene plus 1 kb upstream and downstream. Genes with summed PCIP greater than 0.01 were reported in Supplementary Data 6.

### Workflow

The pipeline of this work was implemented with Snakemake 7.32.4 [57]. The scripts, snakefiles, working conda environment definitions in yaml format, and the dependency analytics graph (DAG) and the rulegraph of the pipeline, are available on 10.5281/zenodo.19386507.

## Results

### Phenotypical characteristics

Descriptive statistics for phenotypes in all generations are shown in Table 1. A smaller mean number of eggs laid (11.9) and a larger coefficient of variance (CV % at 76.1%) were observed during early period of EN measurement (EN1; 20 w – 23 w), while during later periods (EN2 – EN13) the mean number of eggs laid ranged from 21.7 to 25.7 and CV % ranged from 17.2% to 35%. During all EN measurements there were always some individuals (from 0.8% to 19.6% of the population) with none egg laid. From 1.8% to 39.7% of the population laid 28 eggs across the periods. The mean weight and CV % for BW at 32 weeks were 1786.2 g and 11.6%, respectively. For EW measured at different weeks, their CV % were similar average at 7.7%, the mean egg weight was increasing from 57.7 to 64.0 g as the hens aged.

**Fig. 2:**
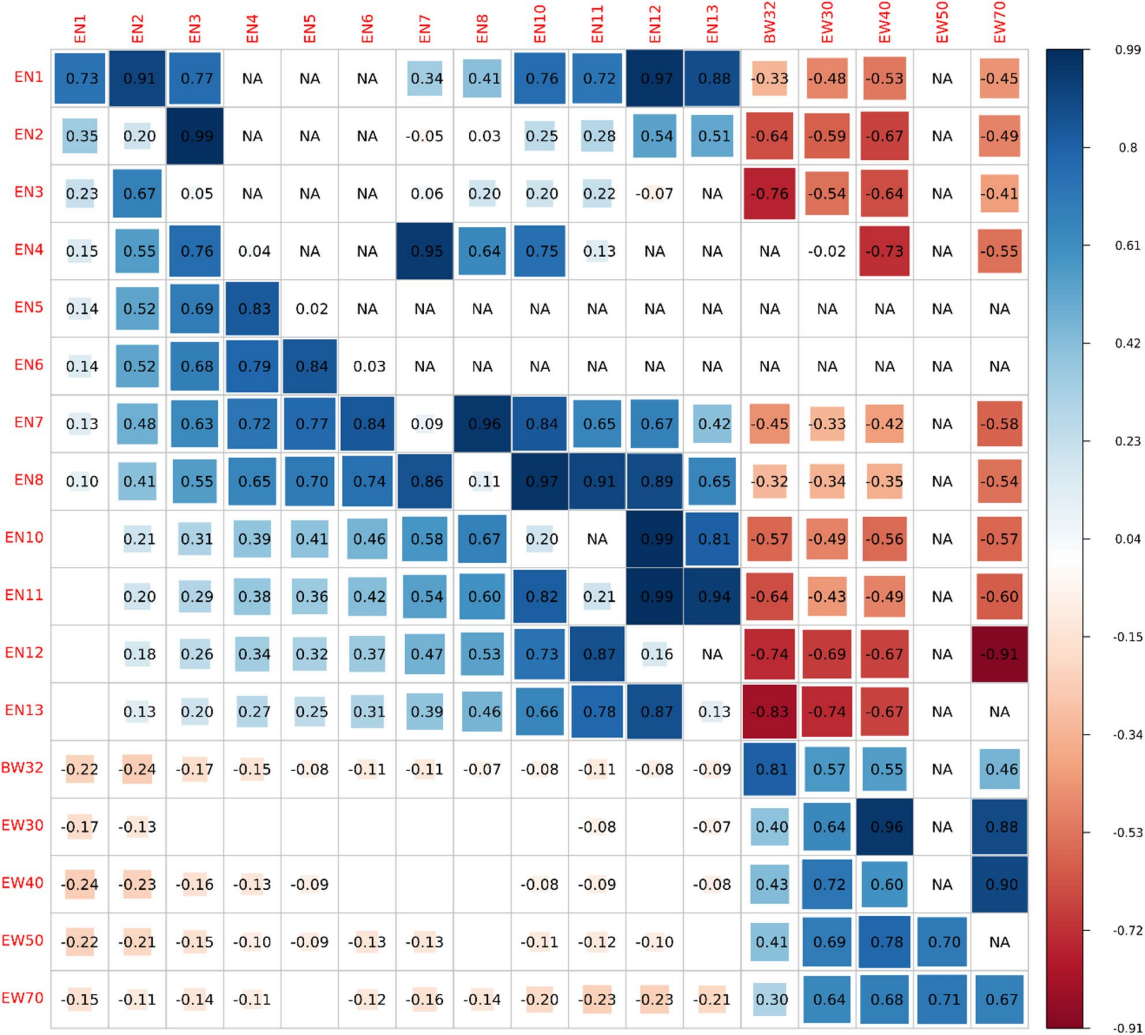
Visualization of genetic (upper triangle, based on imputed genotypes) and phenotypic (lower triangle) correlations between traits after adjusting for generation. Genetic correlation estimates that did not converge were marked as “NA”. Pearson’s correlations were calculated from phenotypic records and statistically insignificant (*p*-values > 0.05) results were blanked. Diagonal shows the estimated *h*^*2*^ for each trait from imputed data

Visualization of the phenotypic correlations (r_p_, lower triangle) and genetic correlations (r_g_, based on imputed SNPs, upper triangle) among all traits, after adjusting for generation effects, together with their estimated heritability, is presented in Fig. 2.

Overall, r_p_ showed moderate to strong positive correlations among EN measurements, although these correlations weakened as the interval between measurements increased. In contrast, many r_g_ across EN failed to converge after adjusting for generation effects, specifically from EN4 to EN6. This period coincided with peak laying performance, during which reduced variance limited reliable estimation. Interestingly, EN1 exhibited strong positive r_g_ with the start and end of the laying period, and moderate positive r_g_ with the mid-to-late laying phase. A similar though weaker pattern was also observed for EN2.

Generally, EN showed negative correlations with both EW and BW in our analysis, with r_g_ showing stronger correlations than r_p_. For EW70, correlations with EN gradually strengthened over the laying period, while for BW32, EW30 and EW40, both r_g_ and r_p_ were strongest at the beginning and end of the laying period, and weakest during the mid-to-late phase.

EW measurements at different weeks of age were consistently and positively correlated, with r_p_ ranging from 0.64 to 0.78 and r_g_ from 0.88 to 0.96. However, r_g_ estimates involving EW50 did not converge. BW32 also showed positive correlations with EW traits, with the strongest r_p_ observed with EW40 (0.43) and the strongest r_g_ with EW 30 (0.57).

Estimated genetic *h*^*2*^ was exceptionally high for EN1 that reached 0.73, and dropped sharply for EN2, EN7–EN13, ranging from 0.09 to 0.21, and was close to zero for other EN periods. In contrast, BW32 had the highest *h*^*2*^ at 0.81, and EW30–EW70 also showed high values, ranging from 0.60 to 0.67.

### Comparison between array and imputed GWAS

All array and imputed GWAS results across traits and groups are presented as Manhattan plots in Supplementary Figs. 1.

**Fig. 3:**
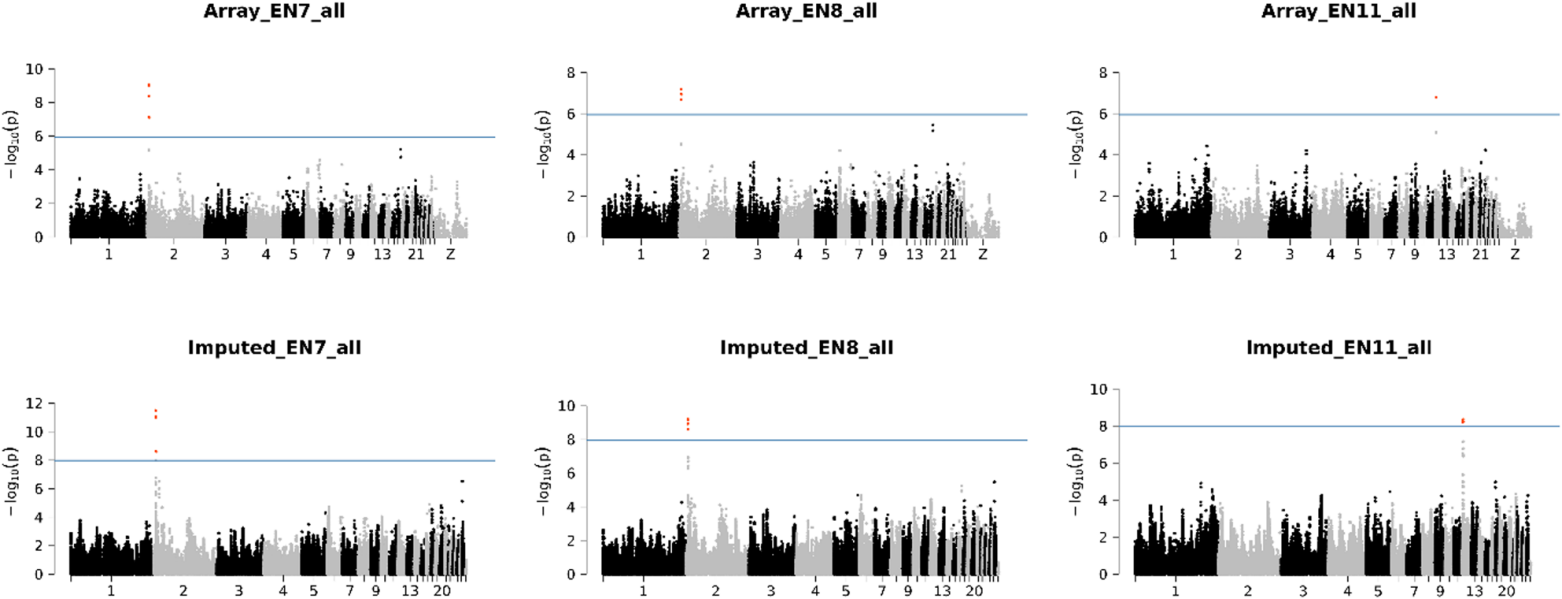
Manhattan plots of single-trait genome-wide association from individuals of all generations for egg number at mid-to-late stages (EN7, 44w–48w; EN8, 48w–52w; EN10, 56w–60w), using array genotypes (top) and imputed genotypes (bottom). Chromosomes are on the x-axis and -log_10_(*P*) on the y-axis. The horizontal blue line indicates the Bonferroni significance threshold. Variants passing the threshold were highlighted in bright colour

Both array and imputed GWAS indicated a polygenic architecture for EN measurements across different sample groups, with many loci each contributing small effects. Some spurious significant variants were observed in the “FounderBC1” and “FounderBC2” groups, particularly during the mid-to-late laying phase (EN7–EN11). However, significant signals were also detected across more sample groups during this same period, most notably on chr 2 and 12 (Fig. 3), with imputed GWAS identifying variants with smaller *p*-values than array GWAS.

Across all EW measurements (EW30, EW40, EW50, and EW70), both array and imputed GWAS consistently identified a significant peak on chr 4 in the “all” and “offspring” groups. For EW30, this chr 4 signal was also significant in the “BC1” and “BC2” groups in the array GWAS, though it did not pass the more stringent significance threshold in the imputed GWAS. In the “IC” group, the signal fell just below the significance threshold in both GWAS. Additionally, a significant signal on chr 1 was detected in the “all” and “offspring” groups in the array GWAS, falling just below significance in the imputed GWAS. A chr 2 signal was significant in the “offspring” group in the array GWAS and showed suggestive significance in the imputed GWAS; it also approached significance in the “all” group in both analyses.

For EW40, the chr 4 signal exceed significance in the “IC” group in the array GWAS, and in the “BC1” group in both GWAS, but was no longer detected in the “BC2” group. The chr 1 signal remained significant in the “all” and “offspring” groups in the array GWAS but did not reach significance in the imputed analysis, while approaching significance in the “BC2” group in the array GWAS.

For EW50, the chr 4 signal was significant in the “IC” group in the array GWAS, but fell below the significance threshold in imputed GWAS, as well as for the “BC1” Group in the array GWAS. At EW70, this signal disappeared entirely in the “IC” group but remained significant in the “BC1” group in the array GWAS. Notably, an emerging signal at a distinct position on chr 1 and a signal on chr 7 were close to the significance threshold in the “BC1” and “BC2” groups, respectively, in the array GWAS.

**Fig. 4:**
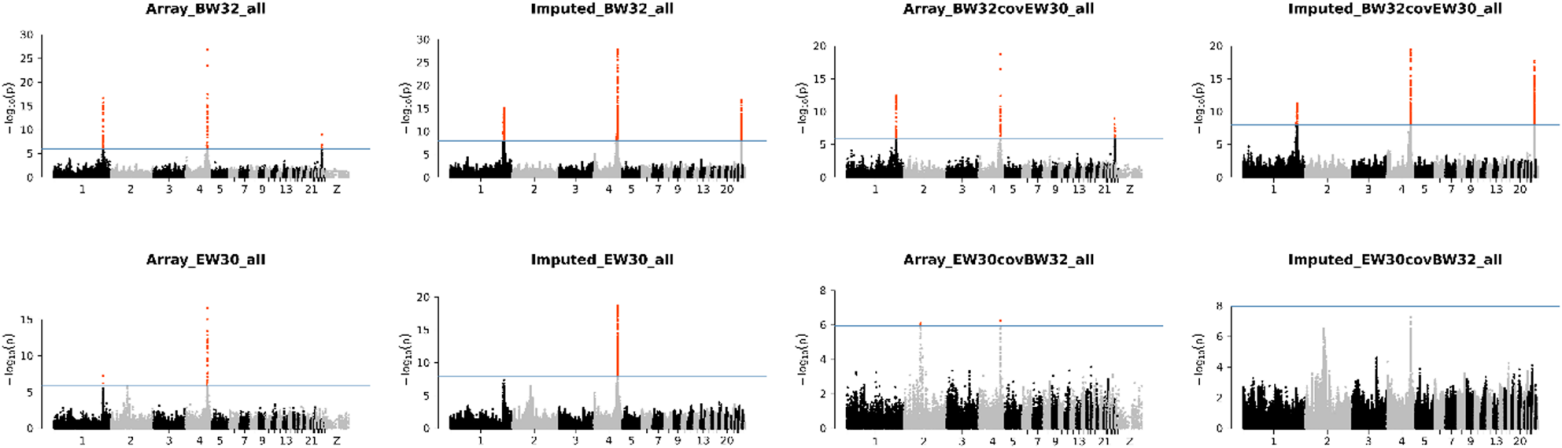
Manhattan plots of single-trait genome-wide association from individuals of all generations, for body weight at 32w (BW32, top) and egg weight at 30w (EW30, bottom), using array genotypes, imputed genotypes, and association including counterpart as a quantitative covariate. Chromosomes are on the x-axis and -log_10_(*P*) on the y-axis. The horizontal blue line indicates the Bonferroni significance threshold. Variants passing the threshold were highlighted in bright colour

For BW32, three major peaks exceeding the significance threshold on chr 1, 4 and 27 were detected by both array and imputed GWAS in the “all,” “offspring,” and “IC” groups. Peaks on chr 1 and 4 were identified in the “BC2” group in both analyses and in the “BC1” group in the array GWAS; only the chr 4 signal remained significant in the imputed GWAS for the “BC1” group, with chr 1 signal falling just below the threshold. Notably, the major peaks on chr 1 and 4 identified for BW32 overlapped with those associated with multiple EW measurements (Fig. 4).

Generally, no significant variants were detected in the WL founders across traits, except for some sporadic signals detected in a few EN traits.

### Multi-trait metaGWAS for EN

**Fig. 5:**
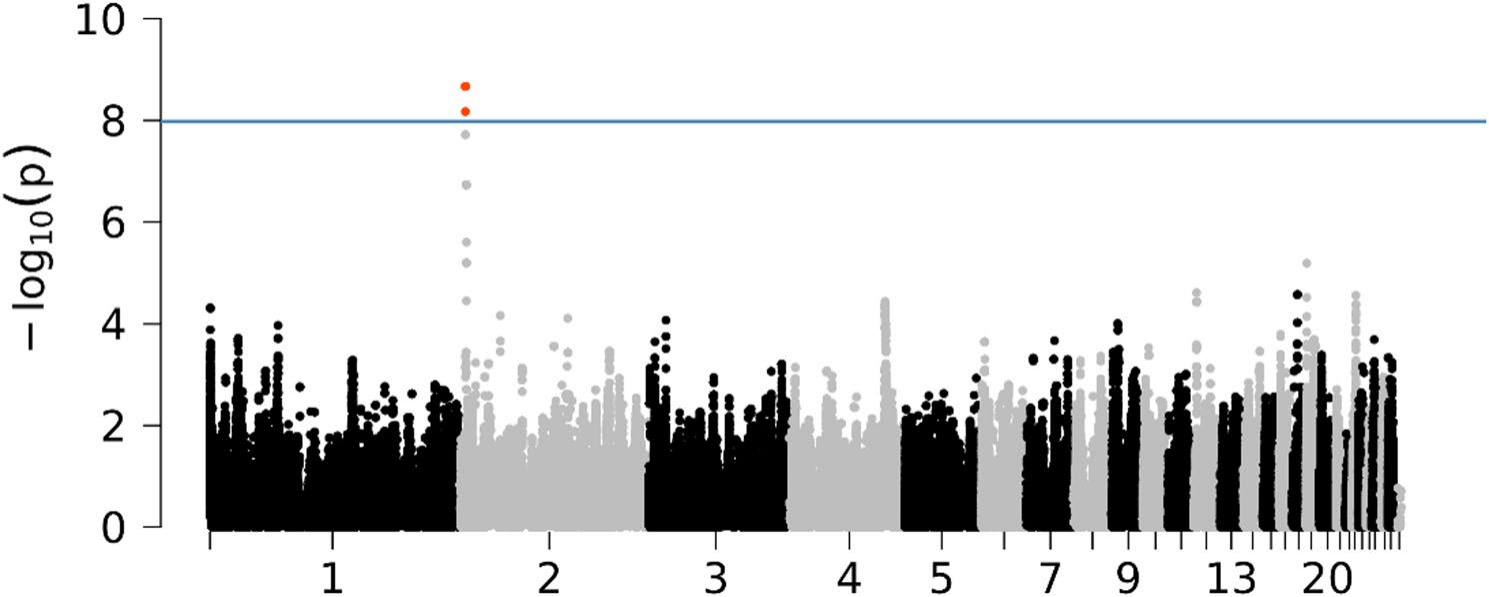
Multi-trait genome-wide association based on results from single-trait associations, including egg number from EN1 to EN13 (EN9 excluded) from individuals of all generations using imputed data. Chromosomes are on the x-axis and -log_10_(*P*) on the y-axis. The horizontal blue line indicates the Bonferroni significance threshold. Variants passing the threshold were highlighted in bright colour

Given the moderate to strong correlations among EN measurements (Fig. 2), and the polygenic background observed for EN in both array and imputed GWAS, we conducted a multi-trait metaGWAS to increase statistical power and identify pleiotropic loci influencing EN across the laying period. The metaGWAS identified several significant variants on chr 2 associated with EN traits from EN1 to EN13 (Fig. 5).

### Including correlated traits as covariates in GWAS

Given the high correlations between BW32 and EW measurements, we performed both array and imputed GWAS for EW traits while including BW32 as a quantitative covariate, and vice versa. This approach provided additional insights into the genetic architecture of these traits.

#### Effect of BW32 covariate on EW GWAS

When BW32 was included as a quantitative covariate in EW GWAS, signals that previously overlapped between BW32 and EW40, EW50, and EW70 on chr 1 and 4 were strongly suppressed and no longer reached significance across any sample group in either GWAS approach. For EW30, covariate adjustment resulted in emerging signals on chr 1 and 4 below the significance, and also helped clarified the presence of the signal on chr 2, which became significant in the “all” group and remained significant in the “offspring” group in array GWAS. Meanwhile, the overlapping signal on chr 1 was strongly suppressed and disappeared across sample groups, whereas the signal on chr 4 showed greatly increased *p*-values and remained just above the significance threshold only in the “all” and “offspring” groups in array GWAS (Fig. 4). Interestingly, for EW70, the previously suggestive signal on chr 1 at a distinct position in the “BC1” group persisted with comparable *p*-values, remaining just below the significance threshold.

#### Effect of EW covariate on BW32 GWAS

Conversely, including EW measurements as covariates in the GWAS for BW32 generally did not eliminate major significant signals on chr 1, 4, and 27 in the “all” (Fig. 4) and “offspring” groups, although a reduction in statistical power was observed (increased *p*-values) on chr 1 and 4 signals across sample groups and different EW covariates. This reduction occasionally prevented these signals from reaching genome-wide significance. For example, in the “IC” group in imputed GWAS across all EW covariates, only the chr 27 signal retained comparable *p*-values and exceeded significance, whereas the chr 4 signal was only significant when EW70 was included as the covariate, and chr 1 signal was strongly suppressed for this sample group. The chr 1 signal dropped below significance when EW50 was included as the covariate in imputed GWAS across all sample groups (noting that EW50 was not recorded in the “BC2” and “FounderBC1” groups). Across different EW covariates, the chr 1 and 4 signals were generally suppressed in the “BC1” and “BC2” groups.

All array and imputed GWAS results including covariate among groups between BW32 and EW30, EW40, EW50, EW70 are available as Manhattan plots in Supplementary Figs. 2.

### Sensitivity of fine-mapping methods in our data

To access the sensitivity to window size of the two fine-mapping methods, we compared lead SNP selection across varying QTL window sizes for selected traits (BW32, EW30, EW70, EN1, EN13) including all generations. For each trait, we tested the windows extended by 1 Mb and 2 Mb upstream and downstream of the original boundaries.

BFMAP-FS consistently selected the same lead SNP across window sizes for *QTL-chr1*, *QTL-chr4*, and *QTL-chr27* of BW32; for *QTL-chr4* of EW30 and EW70; and for *QTL-chr2* of EN1, with comparable log_10_(BF) and PCIP values. In contrast, for EN13, BFMAP-FS results varied: the lead SNPs identified from extended windows were located 2.0 Mb and 2.4 Mb away from the original lead SNP, along with higher log_10_(BF) and comparable PCIP values.

FINEMAP-sss selected the same lead SNP across window sizes only for *QTL-chr4* of EW30. For weight measurements, the distance between lead SNPs across window sizes ranged from 86 bp to 23.6 kb, indicating relatively small variation, whereas for EN traits the differences were larger, ranging from 1 Mb to 3.2 Mb. Detailed results are provided in Result 3 of Supplementary Data 2.

### Fine-mapping GWAS-identified QTLs and enrichment analysis

Fine-mapping analysis was conducted using BFMAP-FS and FINEMAP-sss to refine QTL regions identified through imputed GWAS in the “all” group, with the aim of pinpointing potential causal variants and candidate genes. EN traits exhibited polygenic signals with no clear peaks, making QTL identification unreliable form single-trait GWAS. Given the moderate to strong correlations among EN measurements, a multi-trait metaGWAS was performed to detect pleiotropic loci associated with EN1–EN13. Fine-mapping was subsequently applied to the significant loci identified in this meta-analysis. Fine-mapped results for 19 QTLs of 17 traits are summarised in Result 4 of Supplementary Data 2, and Supplementary Data 3 and 5. Additional results generated by BFMAP-FS can be found in Supplementary Data 4 and 6.

#### Body weights at week 32

For BW32, three QTLs were identified on chr 1, 4, and 27.

#### QTL-chr1

FINEMAP-sss identified two competing models, with posterior probabilities of 0.59 and 0.41 under assumptions of one and two causal SNPs, respectively. The first CS showed high mean absolute LD of 0.96 and a log_10_(BF) of 11.1. The second CS showed moderate LD (0.37 and 0.30) with lower log_10_(BF) (5.4 and 4.9). In total, FINEMAP-sss identified 1,459 variants with PIP > 0, of which 60 retained log_10_(BF) ≥ 2; the maximum marginal PIP was 1%. Twelve genes had summed PIP > 1%, with *FAM124A* showing the highest at 10.3%.

BFMAP-FS estimated regional *h*^*2*^ at 0.21 and identified a single signal confined to a narrower region. After signal filtration (*P* < 5 × 10⁻⁵), the signal was retained, comprising 245 variants in the CS. BFMAP-FS highlighted 7 genes, 4 of which overlapped with FINEMAP-sss results (*FAM124A*, *WDFY2*, *FOXO1*, *INTS6*). Among these, *ENSGALG00000052822* showed the highest PCIP value (48.2%), while the remaining genes had substantially lower PCIP values (1.4—9.2%). Four *3 prime UTR variants* showed increased PCIP after enrichment, rising from 1.1% to 23.2%. Under the “Impact” category, two additional variants showed a more pronounced increase, with PCIP rising from 0.7% to 47.5%, though this did not substantially affect the gene-level PCIP. After enrichment under the “Consequence” category and “Biotype” categories, the PCIP of *ENSGALG00000052822* increased to 93.0% and 52.8%, respectively; only this gene and *WDFY2* (which decreased from 5.0% to 2.0%) retained PCIP > 1%.

#### QTL-chr4

FINEMAP-sss identified two competing models with posterior probabilities of 0.85 and 0.15. The first CS showed high mean absolute LD of 0.99 and a log_10_(BF) of 25.2. The second CS showed differing LD (0.99 and 0.30) with log_10_(BF) of 27.2 and 4.15. FINEMAP-sss identified 786 variants with PIP > 0, of which 94 retained log_10_(BF) ≥ 2; the maximum marginal PIP was 0.96%. Two genes—*NCAPG* and *LCORL*—had summed PIP of 70.7% and 68.9%, respectively.

BFMAP-FS estimated regional *h*^*2*^ at 0.43 and identified a single signal near the *NCAPG*—*LCORL* region and its upstream area, retained after filtration with 107 variants. Enrichment analysis was performed on combined CS from BW32 and EW traits. Two variants at 75,870,903 bp and 75,871,052 bp showed significantly increased PCIP (from 1.0% to 39.3%) post-enrichment under the “Consequence” category, annotated as *3 prime UTR variant*. At the gene level, *NCAPG* and *LCORL* displayed high PCIP values of 74.7% and 72.6%, with slight improvement after enrichment.

#### QTL-chr27

FINEMAP-sss identified two competing models with posterior probabilities of 0.73 and 0.27. The first CS showed high mean absolute LD of 0.98 and a log_10_(BF) of 11.6. The second CS showed low LD (0.21 for both SNPs) with log_10_(BF) of 4.34 and 4.29. FINEMAP-sss identified 2,391 variants with PIP > 0, of which 50 retained log_10_(BF) ≥ 2; the maximum marginal PIP was 3.2%. Five genes had summed PIP > 1%, including *IGF2BP1* (55%), *GIP* (23.9%), *SNF8* (16.8%), *UBE2Z* (10.8%) and *KANSL1* (1.81%).

BFMAP-FS estimated regional *h*^*2*^ at 0.37 and identified one signal retained after filtration with 42 variants. *IGF2BP1* consistently showed the highest PCIP values (68.3% pre-enrichment; 76.2% and 70.8% post-enrichment under “Consequence” and “Impact” categories, respectively). Additional genes—*GIP*, *SNF8*, *UBE2Z*, and *ENSGALG00000001525*—were also identified, with PCIP values ranging from 2.7% to 15.5% across categories before and after enrichment.

#### Egg Weight (EW)

For all EW measurements, one QTL was identified on chr 4.

FINEMAP-sss identified two competing models across EW at different times points. The posterior probability for the first model increased with age (0.29, 0.57, 0.62, 0.90), with log_10_(BF) ranging from 7.78 to 12.7 and high mean absolute LD (> 0.97 for all except EW50, which was 0.78). Correspondingly, the posterior probability for the second model decreased with age, with log_10_(BF) ranging from 3.67 to 10.6 and mean absolute LD values of 0.46–0.99 for the first causal SNP and 0.31–0.37 for the second causal SNP. Across EW traits, the number of variants with PIP > 0 ranged from 1,038 to 2,863, while 17–21 variants retained log_10_(BF) ≥ 2, with maximum marginal PIP of 3.61%. FINEMAP-sss consistently identified *NCAPG* (PIP: 11.7–46.8%), *LCORL* (8.7–41.1%), *LDB2* (5.5–40.1%), and *TAPT1* (1.3–3%) across four EW measurements. Additionally, *PROM1* (2.4–14.4%) and *SLIT2* (2.9– 9.7%) were identified for all EW except EW70, and *CD38* (2.9%) was identified only for EW30.

BFMAP-FS estimated regional *h*^*2*^ decreasing with age, ranging from 0.73 to 0.21. Enrichment analysis on *QTL-chr4* was performed using combined CS from BW32 and EW traits, as the limited number of retained variants per trait precluded reliable estimation. Enrichment estimation was not feasible for the “Impact” category due to a lack of annotation variability.

For EW30, two signals were detected and retained after filtration: one localized near *SLIT2* and *LDB2*, and another in an intergenic region. The combined signal comprised 444 variants. An *intergenic variant* located at 75,309,616 bp showed increased PCIP after enrichment, rising from 0.9% to 95.0%, though no genes retained with PCIP > 1% after enrichment under the “Consequence” category. Overall, variants highlighted *LDB2*, *SLIT2*, *ENSGALG00000054285*, and *ENSGALG00000032384*, with PCIP values remaining unchanged after enrichment under the “Biotype” category (PCIP: 65.4%, 28.7%, 10.1%, and 1.3%, respectively).

For EW40, a single signal was retained after filtration, localized near the *NCAPG*—*LCORL* region as two clusters (199 variants). Notably, two *3 prime UTR variants* increased in PCIP after enrichment under the “Consequence” category, rising from 0.6% to 24.1%. Pre-enrichment, *NCAPG* (46.1%) and *LCORL* (40.8%) were highlighted. After renormalization under the “Consequence” category, the PCIP of *NCAPG* increased slightly to 51.8%, while that of *LCROL* dropped below 1%. Under the “Biotype” category, PCIP values for *NCAPG* and *LCORL* were 24.5% and 18%, respectively.

For EW50, one signal was retained after filtration, localized near the *NCAPG*—*LCORL* and *LDB2* region as two clusters, comprising 155 variants. Pre-enrichment PCIP values highlighted *NCAPG* (4.3%) and *LDB2* (4.8%). After renormalization under the “Consequence” category, the PCIP of *NCAPG* increased minorly to 4.5%, while that of *LDB2* dropped below 1%. Under the “Biotype” category, PCIP values for *NCAPG* and *LDB2* were 4.4% and 1.5%, respectively.

For EW70, one signal was detected overlapping the EW40 and EW50 locus, comprising 172 variants. Pre-enrichment, these variants highlighted *NCAPG* (7.3%), *LCORL* (7.1%), and *LDB2* (4%). Similar to EW50, only *NCAPG* remained after enrichment under the “Consequence” category, with renormalized PCIP increased to 9.7%. Under the “Biotype” category, PCIP values for *NCAPG*, *LCORL*, and *LDB2* were 2.4%, 2.4%, and 1.3%, respectively.

#### Egg Number (EN)

The multi-trait metaGWAS for EN1–EN13 identified one QTL on chr 2.

FINEMAP-sss identified a single model for most EN traits, with posterior probability of 1 for EN1–EN3, EN5, EN6, and EN11–EN13. For EN8, the posterior probability was 0.95, and for EN4 and EN7, two models were identified with negligible posterior probabilities for the second model (0.07 and 0.05). The mean log_10_(BF) across EN traits was 5.5. Mean absolute LD within CS was 0.33 on average, except for EN4, EN7, and EN8, where LD was near 1. Across EN traits, the number of variants with PIP > 0 ranged from 1,564 to 13,115, while only EN4–EN8 had variants retained with log_10_(BF) ≥ 2 (35–38 variants), with maximum marginal PIP of 2.7%; no variants met this threshold for other EN traits. The retained variants overlapped with CS identified by BFMAP-FS for these traits.

FINEMAP-sss highlighted 18 genes across EN traits. *GARS* was identified by all EN results, with high PIP for EN4–EN8 (> 42.2%) and lower PIP for other EN measurements (2.1–16.5%). PIP values for other genes across EN traits ranged from 1% to 8.8%. *CRHR2* was highlighted by all EN traits except EN5 and EN8; *VILL* was identified for EN10, EN12, EN13; *NBEAL2* and *KLHL18* were identified uniquely for EN1; and additional 13 genes were frequently highlighted during the beginning and ending of the laying period;

BFMAP-FS estimated *h*^*2*^ for the *QTL-chr2* across EN2–EN13 as generally low, ranging from near 0 to 0.1, though it reached 0.49 for EN1. CS sizes ranged from 21 to 409 variants before filtration. BFMAP-FS highlighted 14 genes, 7 of which overlapped with FINEMAP-sss results. *PLCD1* showed the highest PCIP (82.5%) for EN3. *GARS* was identified across EN3–EN13, with PCIP ranging from 22.9% to 58.5%. *ENSGALG00000049955* was also identified across EN2–EN13 (PCIP 1.7–27.8%). Five genes were detected specifically for EN2: *CRHR2* (9.9%), *MYD88* (7.1%), *DLEC1* (3.6%), *CTDSPL* (1.8%), and *ENSGALG00000049540* (1.6%). Another five genes were identified during both the beginning and ending of the laying period: *ENSGALG00000005884*, *OXSR1*, *PLCD1*, and *XIRP1* (PCIP 1.8–82.5%). Additionally, *ENSGALG00000005934* was specific to EN1 (4.9%) and EN2 (2.1%); *ENSGALG00000049302* and *ENSGALG00000050406* (PCIP 1.1–3.7%) were specific to later stages.

After signal filtration, CS sizes were reduced to 21–38 variants, with no variants retained for EN2 and EN10–EN13. Enrichment analysis on *QTL-chr2* was performed using combined CS from all EN traits to enhance maximum likelihood estimation. Following enrichment, *GARS* and *ENSGALG00000049955* continued to be highlighted across EN3–EN8. *GARS* showed increased PCIP under the “Impact” category (59.1–63.8%), though reduced PCIP under “Consequence” (22.5–22.9%), and was not identified under “Biotype”. In contrast, *ENSGALG00000049955* showed increased PCIP highest under “Biotype” (95.7–97.4%), followed by “Consequence” (73.2–74.5%), and slight decreased under “Impact” (22.3–24.1%). Notably, *ENSGALG00000005934* and *XIRP1* retained and uniquely identified in EN1 after enrichment, with PCIP decreasing from 4.8% to 3.8% and 2.3% under the “Consequence” and “Impact” categories for *ENSGALG00000005934*, and PCIP increasing from19.4% to 60.0% under the “Impact” category for *XIRP1*.

## Discussion

### Enhancing GWAS through backcrossing generations and imputation

A backcrossing scheme involves the introduction of multiple recombination events that can break down long-range LD blocks, potentially improving QTL mapping resolution and refinement [23]. In this study, GWAS in the “IC” generation identified more and stronger signals than in other generations individually, while analyses combining all generations benefited from a larger sample size and identified a greater number of QTLs compared to single-generation analyses. Furthermore, imputation of array-genotyped data substantially increased the density of variants across the genome, which enhanced the statistical support of QTLs that were already identified in array GWAS, although the stricter significance threshold meant that not all signals reached genome-wide significance in the imputed analysis. These findings suggest that combining breeding strategies with advanced genomics tools can improve the resolution and detection power of GWAS in structured populations.

### Including correlated traits as covariates in GWAS reveals pleiotropic and trait-specific loci

Including covariates in GWAS can help account for confounding factors that may obscure true genetic signals. Specifically, when two traits are correlated, conditioning GWAS on one trait by including it as a covariate can help to disentangle their shared and distinct genetic associations. In this study, BW32 and EW traits exhibited strong phenotypic and genetic correlations across different time points. To explore the genetic relationships between these traits, we performed GWAS for EW traits while including BW32 as a quantitative covariate, and vice versa.

When BW32 was included as a covariate in the GWAS for EW at four time points, the previously identified QTLs on chr 1 and 4 were substantially attenuated or disappeared entirely. This observation suggests that these associations may reflect shared genetic effects between BW32 and EW, rather than effects specific to EW. In contrast, when EW traits were included as covariates in the GWAS for BW32, the coinciding signals on chr 1 and 4 generally remained significant, albeit with reduced statistical power (i.e., larger *p*-values). This pattern indicates that, while BW32 and EW shared genetic components at these loci, BW32 may retain additional genetic variation at these regions that is independent of EW. The locus on chr 27 appeared largely unaffected by EW adjustments, suggesting that its association with BW32 is statistically independent of EW. In some cases, covariate adjustment helped clarify spurious association patterns, for example, the GWAS for EW70 when BW32 was included as a covariate revealed stability of *p*-values for suggestive signals. However, these findings provide statistical evidence consistent with pleiotropy rather than direct proof of functional effects. Further investigation to explore the biological mechanisms for these loci is needed to provide valuable insights determining whether they represent true genetic effects or arise from statistical independence in the data.

These findings illustrate the value of including correlated traits as quantitative covariate in GWAS to help distinguish between pleiotropic and trait-specific associations, thereby refining association signals and enhancing the interpretability of GWAS results.

### Variation in heritability estimates

The variation in *h*^*2*^ estimates observed at both the genome-wide and QTL level in this backcrossing scheme reflects the complex genetic architecture underlying the studied traits in a heterogeneous population. Overall, *h*^*2*^ estimates were higher for BW32, EW at different time points, and EN during the early stage of production (highest for EN1, moderate for EN2). In contrast, *h*^*2*^ estimates approached zero during the peak laying period (EN3—EN6), then increased again during the mid-to-late stages (EN7—EN13, Fig. 2). This decline in *h*^*2*^ with advancing age has been previously reported in chickens and may reflect the increasing influence of environmental factors on egg production over time [58, 59]. Our findings partially support this interpretation, as we observed a slight increase in *h*^*2*^ from EN7 onwards, reaching moderate levels by EN10. Furthermore, the relatively stable *h*^*2*^ estimates for EW across ages observed in our data align with previous reports that environmental variation in EW does not change substantially over time [60].

Interestingly, GWAS results revealed more suggestive or significant variants during periods of low-to-moderate *h*^*2*^ (EN7—EN11), while a highly polygenic architecture was observed for EN1, which had the highest *h*^*2*^ (0.73). Additionally, the QTL on chr 2 derived from the multi-trait metaGWAS explained a substantial proportion of variance for EN1 (*h*^*2*^ = 0.49) but not for other EN traits. This pattern may reflect differences in genetic architecture of EN across the laying cycle, with early production influenced by many small-effect loci and later stages potentially affected by fewer loci alongside with environmental factors.

For EW30, the regional *h*^*2*^ estimate for *QTL-chr4* (0.73) exceeded the genome-wide *h*^*2*^ estimate (0.64). This may reflect regional haplotype structure and linkage disequilibrium in the backcross population, which can lead to inflated variance estimates when the QTL-based GRM captures not only the QTL effects but also part of the background covariance due to ancestry. In addition, BFMAP’s eigendecomposition approach can be sensitive to low‑rank GRMs, such as those built from small QTL regions. Such sensitivity may be more pronounced when the true genetic signal is strong, as the few dominant eigenvalues then disproportionately influence the variance partition. Because standard errors are unavailable, the statistical significance of this difference cannot be tested. In contrast, QTL *h*^*2*^ estimates for EW40, EW50, and EW70 were substantially lower and remained moderately influential through later stages (> 0.2). This decline is consistent with decreasing PIP and PCIP values observed for identified genes in later EW measurements, indicating that the contribution of this QTL to EW may diminish with age. Moreover, the attenuation of GWAS signals for EW when BW32 was included as a covariate was most pronounced at later EW stages, suggesting that the statistical association of this QTL becomes increasingly dependent on BW32 with age. Whereas for BW32, *QTL-chr4* and *QTL-chr27* persisted in GWAS with EW covariates and explained comparable proportions of variance (*h*^*2*^ ≈ 0.4), while *QTL-chr1* explained slightly less (*h*^*2*^ = 0.21) and fell below the significance threshold when EW50 was included as a covariate. This pattern further supports the pleiotropic nature of QTLs on chr 4 for BW and EW traits. Finally, the varying sample sizes may affect the precision of *h*^*2*^ estimates, given that generations of “FounderBC1” and “BC2” did not have data for EW50. Nevertheless, QTL-based and genome-wide *h*^*2*^ estimates were strongly correlated (Pearson’s *r* = 0.78), suggesting that the QTL-based estimates partially reflect trait genetic architecture.

### Fine-mapping identifies promising candidate genes associated with multiple traits

Fine-mapping analyses using BFMAP-FS and FINEMAP-sss identified several overlapping candidate genes with statistical support for association across multiple traits. The two methods differed in their output: FINEMAP-sss identified a larger number of genes (42 genes) but many with generally low PIP values (median 2.2%), whereas BFMAP-FS provided additional functionality through functional annotation enrichment to refine candidate gene lists (17 genes after refinement, median PCIP 23.8%). These differences reflect the distinct algorithms underlying each method, as well as their differing sensitivity to QTL window definition.

Many fine-mapping methods, such as FINEMAP, were developed primarily for population of unrelated individuals (e.g., human population) and may perform less reliably in livestock populations [61]. Fine-mapping in livestock populations is challenging due to extensive LD and limited effective population size. This expectation is consistent with our sensitivity analysis, where FINEMAP-sss showed higher sensitivity in lead SNP selection across varying window sizes. In contrast, BFMAP-FS, which is designed for populations of related individuals, demonstrated greater stability to window sizes. Therefore, while we reported results from both methods to provide a comprehensive overview, we suggest placing a greater emphasis on the BFMAP-FS results.

The generally low PIPs and PCIPs observed for most candidate genes in this study reflect the limited power of fine-mapping in such populations; consequently, enrichment analyses from BFMAP-FS were feasible only after pooling variants across related traits. These patterns are consistent with the expectation that fine-mapping in livestock is inherently underpowered compared to human studies. Despite these methodological differences, both methods highlighted several genes with plausible biological relevance to the traits studied.

#### NCAPG and LCORL

*NCAPG* and *LCORL* were consistently highlighted for both BW32 and all EW measurements, with comparatively high PIP and PCIP values. *NCAPG* encodes a subunit of the condensin complex involved in chromosomal regulation during mitosis and meiosis, while *LCORL* encodes a ligand-dependent nuclear receptor corepressor-like protein involved in transcriptional regulation via RNA polymerase II. Both genes have been extensively linked to growth traits across species. In cattle, the *NCAPG*–*LCORL* locus is robustly associated with growth, feed intake, and carcass traits, as supported by genomic association studies, gene expression analyses, and haplotype mapping [62–64]. In horses, this locus influences body size and conformation traits with strong GWAS signal [65, 66]. Selective sweep analysis has shown strong selection signature of *LCORL* associated with stature in pigs [67]. Recently, Bai et al. confirmed causal mutations in the *NCAPG*–*LCORL* region affecting body weights, through combined analyses of selective sweep across domesticated species and gene-editing studies in mice [68].

Although *NCAPG* and *LCORL* have well-established roles in growth, direct biological evidence linking them to egg weight is limited. In our study, PIP and PCIP values for these genes were consistently lower for EW than for BW, and declined sharply with increasing age. For instance, while PIP and PCIP values for EW40 exceed 40%, they dropped to < 15.3% and < 11.7% in EW50 and EW70, suggesting a diminishing statistical contribution. Notably, these two genes were not identified by BFMAP-FS for EW30, although FINEMAP-sss assigned them with PIP values above 11.5%. Taken together with the strong phenotypic and genetic correlations observed between BW and EW, and the loss of GWAS signals when BW32 was included as a covariate, these findings suggest that the statistical associations of *NCAPG* and *LCORL* with EW may be partially mediated through their effects on body weight.

#### *IGF2BP1* and other genes on chr 27 for BW32

Fine-mapping of BW32 highlighted *IGF2BP1* (insulin-like growth factor 2 mRNA-binding protein 1), a gene associated with growth and body size across various livestock species [69–73]. In chickens, causal promoter variants in *IGF2BP1* regulating body size were identified via pan-genome analysis, particularly between 3 and 12 weeks of age [74]. Additionally, *IGF2BP1* has been shown to promote fat and muscle development in chickens, especially at 8 weeks and 120 days, respectively [75, 76]. Whereas it’s expression level has been reported to decline with age in poultry [71, 76, 77]. Interestingly, *IGF2BP1* alone has been reported to inhibit cell proliferation unless coupled with post-translational modification, suggesting that its biological activity may be context-dependent [77]. In our study, BW was measured at 32 weeks of age, which is later than in previous studies, yet *IGF2BP1* was still consistently identified with high PIP and PCIP values, with PCIP increasing by 9.8% after functional enrichment. Thi finding suggests continued statistical support for *IGF2BP1* and its potential long-term impact in influencing body weight.

Apart from *IGF2BP1*, Fine-mapping additionally identified several nearby protein-coding genes, including *GIP* (glucose response), *SNF8* (protein transport), *UBE2Z* (apoptosis); *ENSGALG00000001525* (autophagosome regulation; NCBI gene name for *CALCOCO2*) from BFMAP-FS and *KANSL1* (regulation of mitochondrial transcription) from FINEMAP-sss. These genes have been previously reported to be associated with breast muscle weights [78], abdominal fat percentage [79], and residual feed intake [80] in chicken, suggesting they may contribute to body weight variation through their roles in energy metabolism.

#### Candidate genes on chr 1 for BW32

For BW32, fine-mapping additionally identified several genes on chr 1. BFMAP-FS highlighted *ENSGALG00000052822* with high PCIP values post-enrichment (93% and 52.8% under the “Consequence” and “Biotype” categories, respectively). *WDFY2* was identified by both methods, with a PIP of 6.1% from FINEMAP-sss and a renormalized PCIP of 2% from BFMAP-FS, though it was not retained after BFMAP enrichment. Both methods also identified *FAM124A*, with a PIP of 10.3% and PCIP of 9.2%. These genes have been reported to be associated with body weight in chickens [81, 82], as well as with stature [83] and skeletal muscle production [84]. BFMAP-FS (before enrichment) and FINEMAP-sss also identified additional genes with generally low probabilities (1.4—4.1%), including *ITS6*, *KPNA3*, *FOXO1*, *CAB39L*, and *RCBTB1*. These genes have been reported in other studies associated with bone traits, body weight, and growth in chickens [4, 82, 84–87].

#### Candidate genes on chr 4 for EW traits

On chr 4, fine-mapping identified several genes other than *NCAPG* and *LCORL* associated with EW across different periods, including *LDB2* (by both methods), *SLIT2*, *TAPT1*, and *PROM1* (by FINEMAP-sss). Additional genes were identified specifically for EW30: BFMAP-FS identified *SLIT2*, *ENSGALG00000054285*, and *ENSGALG00000032384*, while FINEMAP-sss identified *CD38*. Among these, *LDB2*, *SLIT2* and *TAPT1* have been widely reported in association with growth traits or body weight [3, 12, 24, 73, 82, 85, 86, 88–91], whereas *PROM1*, *CD38* and *ENSGALG00000054285* have been less frequently documented in chickens [88, 89]. Given their moderate to low PIP and PCIP values, taken together the loss of corresponding GWAS signals for EW after accounting for BW, and their biological functions—transcription regulation (*LDB2*), cell signalling and regulation (*SLIT2*, *CD38*,* PROM1*, *TAPT1*)—these loci may contribute to egg weight primarily through pathways related to body growth.

#### Genes associated with EN

For EN, fine-mapping identified 18 genes associated across or within specific laying periods, with 7 overlapping between the two methods. After signal filtration, BFMAP-FS highlighted two genes consistently linked from EN3 to EN8, and two genes specific to EN1. However, these genes generally did not overlap with findings reported from prior studies, in line with the observation that many QTLs identified for egg production in chickens rarely replicated across studies [1, 2, 58]. Nonetheless, their molecular and biological functions can provide insights into the potential mechanisms influencing reproductive traits.

During early and late laying periods, BFMAP-FS identified genes indicated roles in oxidative stress response (*OXSR1*), lipid metabolism (*PLCD1*), actin cytoskeleton organization (*XIRP1*), and protein phosphorylation (*ENSGALG00000005884*). For specific periods, BFMAP-FS identified genes involved in transmembrane transport (*ENSGALG00000005934*, NCBI gene name for *SLC22A13*) for EN1 and EN2; cell surface receptor signalling (*CRHR2*), cell cycle regulation and differentiation via tumour suppressor activity (*CTDSPL*), defence response to tumour cells (*DLEC1*), and Immune signalling (*MYD88*) for EN2. FINEMAP-sss additionally identified genes involved in mRNA splicing (*CCDC12*), vasculogenesis and heart development (*CCM2*), cardiac muscle contraction (*MYL3*), microtubule-based movement (*KIF9*), cell membrane electrical signalling (*SCN11A*), early neural crest development (*SETD2*), and DNA damage and repair (*WDR48*) during early and late laying periods, as well as protein ubiquitination (*KLHL18*) and intracellular protein localization (*KBEAL2*) for EN1, and microskeleton organization (*VILL*) during late phases. Two genes were consistently identified across multiple periods with high PIP or PCIP values. *GARS*, identified by both methods, encodes a glycyl-tRNA synthetase that is essential for protein biosynthesis in both the cytoplasm and mitochondria. *ENSGALG00000049955*, identified by BFMAP-FS, contains non-coding elements and has not been well-characterized.

Together, the biological processes associated with these genes suggest that egg production may be potentially influenced through diverse pathways, particularly those related to cellular development, metabolism, and immune regulation. However, these interpretations are based on statistical associations and functional annotations, experimental validation is needed to establish causality.

### Limitations of imputed-based fine-mapping

Overall, fine-mapping of GWAS-identified QTLs has uncovered promising candidate genes for multiple traits. Nevertheless, it is important to keep in mind that the coverage and quality of imputed genotypes may limit fine-mapping resolution and downstream analyses such as signal filtration by a *p*-value threshold, and functional enrichment. In this study, imputation substantially increased the number of variants from ~ 46.6k to ~ 4.9 M; however, the resulting dataset remains less comprehensive than WGS data. Furthermore, the concordance between imputed and array genotypes did not exceed 96%, which introduces uncertainty. These limitations may affect the ability to detect causal variants, particularly structural or rare variants, despite efforts to extend QTL regions by 1 Mb upstream and downstream to capture more informative markers. These factors should be carefully considered when interpreting fine-mapping results, especially in the context of imputed data.

## Conclusions

In this study, we demonstrated that combining individuals across generations in a backcrossing scheme and applying genotype imputation increased the statistical power and resolution of GWAS for economically important traits in chickens. Moreover, incorporating correlated traits as quantitative covariates in GWAS offered valuable insights that helped distinguish between pleiotropic associations and trait-specific signals.

This study applied two fine-mapping methods to GWAS-identified QTLs in a chicken backcrossing scheme, enabling refinement of candidate regions and identification of candidate genes underlying important traits. Our findings not only confirmed previously reported loci but also highlight additional genes to provide new insights into the complexity of trait inheritance. Nevertheless, the coverage and accuracy of genotype imputation may limit the fine-mapping resolution. Overall, this study illustrates the utility of fine-mapping approaches for refining GWAS signals and generating statistical hypotheses about the genetic architecture of complex traits, offering potential targets for future functional validation and real-life breeding applications.

## Supplementary Information

Supplementary Material 1. Supplementary Figures 1: Combination of Manhattan plots of array and imputed GWAS across traits and groups. Supplementary Figures 2: Combination of Manhattan plots of array and imputed GWAS among groups, including BW32 as a covariate for EW (EW30, EW40, EW50, EW70) and vice versa. Supplementary Figures 3: Principal component analysis (PCA) of reference and target population used for imputation. Supplementary Figures 4: Visualization of phenotypic distribution between generations. Supplementary Data 1: Phenotypic records and generation of individuals. Supplementary Data 2: Result1 – Imputation accuracy across MAF bins (0, 0.01, 0.05, 0.1, 0.3, 0.5) accessed using five-fold cross-validation with two repetitions, including IQS, mean concordance, mean Pearson's correlation, minimum Pearson's correlation, and DR2 for each repetition and fold. Result2 – Mean genomic inflation factors for each trait and sample group combination of array and imputed GWAS. Result3 – Comparison of lead SNP selection on varying QTL windows of selected traits between BFMAP-FS and FINEMAP-sss. Result4 – QTLs of 17 traits for fine-mapping and the number of variants within the QTL region; from BFMAP: estimated QTL heritability and number of variants in the CS before and after signal filtration; from FINEMAP: number of variants with marginal PIP > 0, number of variants with marginal PIP > 0 and log10(BF) ≥ 2, posterior probability, log10(BF) quantifying evidence that there is an additional causal signal, and average absolute correlation between SNPs, for each model assuming the number of causal SNP is 1 or 2. Supplementary Data 3: BFMAP: Fine-mapping pinpointed genes before signal filtration with summed PCIP > 0.01, ordered by chromosomes, descending summed PCIP, and genes. FINEMAP: Fine-mapping pinpointed genes with summed PIP > 0.01, ordered by chromosomes, descending summed PIP, and genes. Supplementary Data 4: Enrichment analysis of BFMAP-FS results with combined traits, including enrichment factor and probability of including causal variants for a functional annotated category, ordered by QTL, category group, and enrichment factor. Supplementary Data 5: BFMAP: CS of BFMAP-FS pinpointed variants after signal filtration with their original and re-normalized PCIP, ordered by trait and re-normalized PCIP in descending order. FINEMAP: variants identified with PIP > 0 and log10(BF) ≥ 2, ordered by trait and PIP in descending order. Supplementary Data 6: Functional enrichment of BFMAP-FS pinpointed genes after signal filtration with summed PCIP > 0.01, ordered by chromosomes, descending summed renormalised PCIP, and genes.

## Data Availability

Restrictions apply to the availability of the genotype data analysed in this study, which is partially owned by Lohmann Breeders (Cuxhaven, Germany) and are available from the authors only with the permission of Lohmann Breeders upon a reasonable request. This study also used a subset of 170 whole-genome sequenced animals from a global reference panel prepared by the Chicken Ge-nomic Diversity Consortium, as reference for the imputation. The reference panel is expected to be made publicly available upon the consortium’s publication. However, Whole-genome sequencing data for the six Araucana founder chickens included in this study are publicly available at the European Nucleotide Archive under accession PRJEB38683.

## Abbreviations

SNP: Single Nucleotide Polymorphism
GWAS: Genome-Wide Association Study
QTL: Quantitative Trait Locus
EN: Egg Number
EW: Egg Weight
BW: Body Weight
LD: Linkage Disequilibrium
WL: White Layer
WGS: Whole-Genome Sequence
DR2: Dosage R-Square (Imputation Quality Metric)
IQS: Imputation Quality Score
GRM: Genetic Relationship Matrix
MAF: Minor Allele Frequency
CS: Credible Set
PCIP: Posterior Conditional Inclusion Probability
VEP: Variant Effect Predictor
CV: Coefficient of Variance
chr: Chromosome
