# Supplementary material for: Bayesian fine-mapping pinpoints candidate genes and pleiotropic loci of production traits from a chicken backcrossing scheme
Source: BMC Genomics. 2026 Jun 16;27:548. doi: 10.1186/s12864-026-13069-z (PMC13270760; doi:10.1186/s12864-026-13069-z)
Supplement: Supplementary file 1 — Supplementary Material 1. Supplementary Figures 1: Combination of Manhattan plots of array and imputed GWAS across traits and groups. Supplementary Figures 2: Combination of Manhattan plots of array and imputed GWAS among groups, including BW32 as a covariate for EW (EW30, EW40, EW50, EW70) and vice versa. Supplementary Figures 3: Principal component analysis (PCA) of reference and target population used for imputation. Supplementary Figures 4: Visualization of phenotypic distribution between generations. Supplementary Data 1: Phenotypic records and generation of individuals. Supplementary Data 2: Result1 – Imputation accuracy across MAF bins (0, 0.01, 0.05, 0.1, 0.3, 0.5) accessed using five-fold cross-validation with two repetitions, including IQS, mean concordance, mean Pearson's correlation, minimum Pearson's correlation, and DR2 for each repetition and fold. Result2 – Mean genomic inflation factors for each trait and sample group combination of array and imputed GWAS. Result3 – Comparison of lead SNP selection on varying QTL windows of selected traits between BFMAP-FS and FINEMAP-sss. Result4 – QTLs of 17 traits for fine-mapping and the number of variants within the QTL region; from BFMAP: estimated QTL heritability and number of variants in the CS before and after signal filtration; from FINEMAP: number of variants with marginal PIP > 0, number of variants with marginal PIP > 0 and log10(BF) ≥ 2, posterior probability, log10(BF) quantifying evidence that there is an additional causal signal, and average absolute correlation between SNPs, for each model assuming the number of causal SNP is 1 or 2. Supplementary Data 3: BFMAP: Fine-mapping pinpointed genes before signal filtration with summed PCIP > 0.01, ordered by chromosomes, descending summed PCIP, and genes. FINEMAP: Fine-mapping pinpointed genes with summed PIP > 0.01, ordered by chromosomes, descending summed PIP, and genes. Supplementary Data 4: Enrichment analysis of BFMAP-FS results with combined trait [file 12864_2026_13069_MOESM1_ESM.zip › Supplementary files/Supplementary Figures 3.pdf]

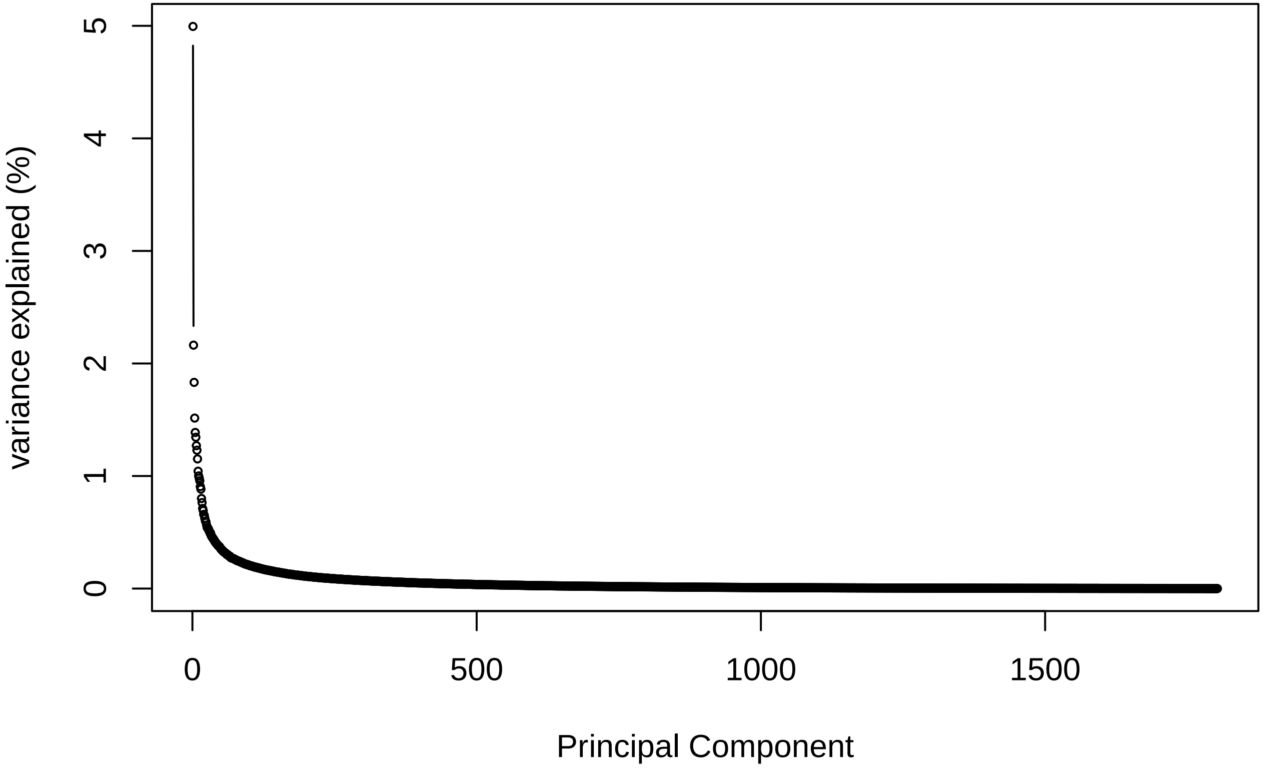
variance explained (%)
0
1
2
3
4
5
0
500
1000
1500
Principal Component

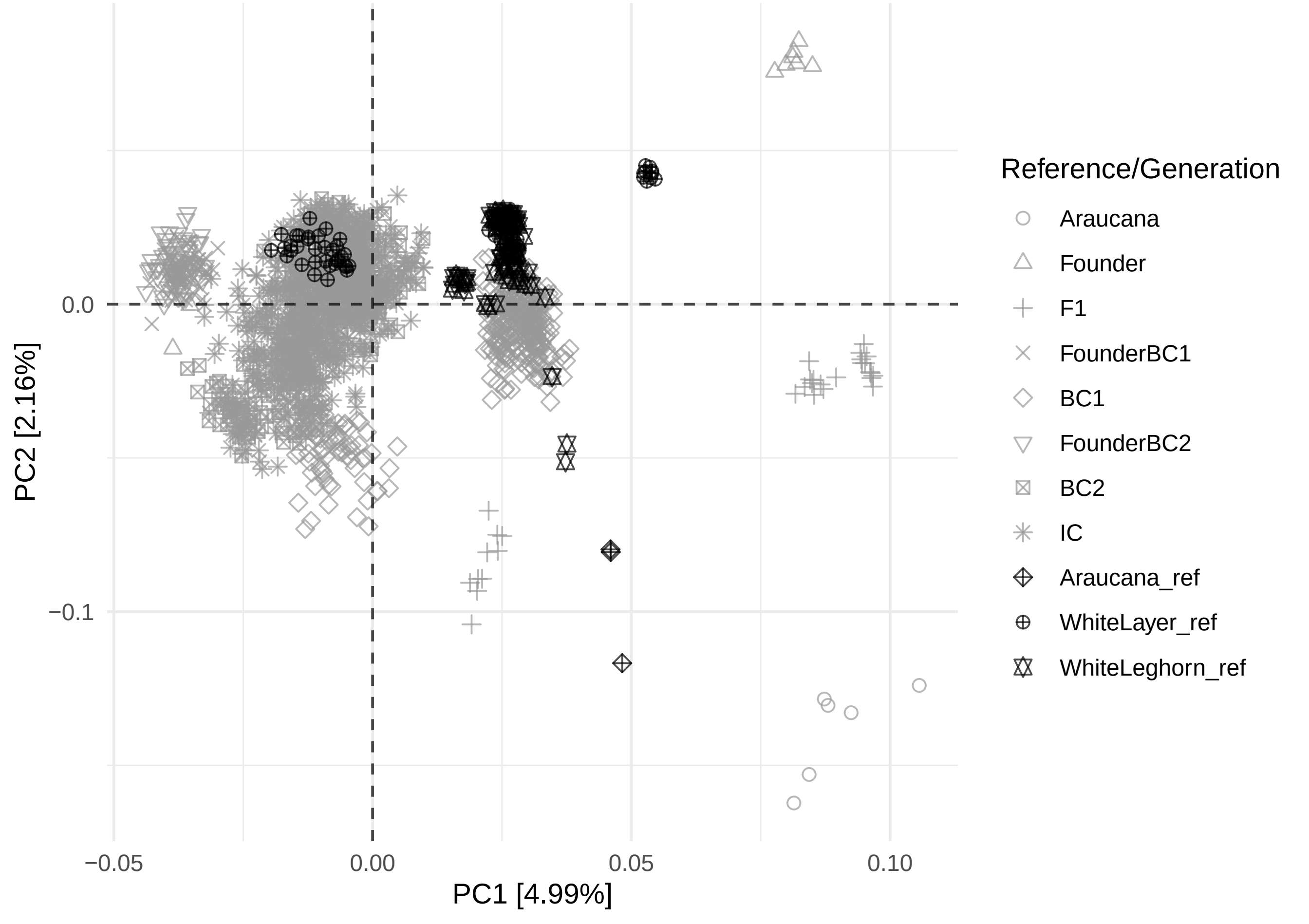

PC1 [4.99%]
PC2 [2.16%]
−0.05
0.00
0.05
0.10
0.0
−0.1
Reference/Generation
Araucana
Founder
F1
FounderBC1
BC1
FounderBC2
BC2
IC
Araucana_ref
WhiteLayer_ref
WhiteLeghorn_ref

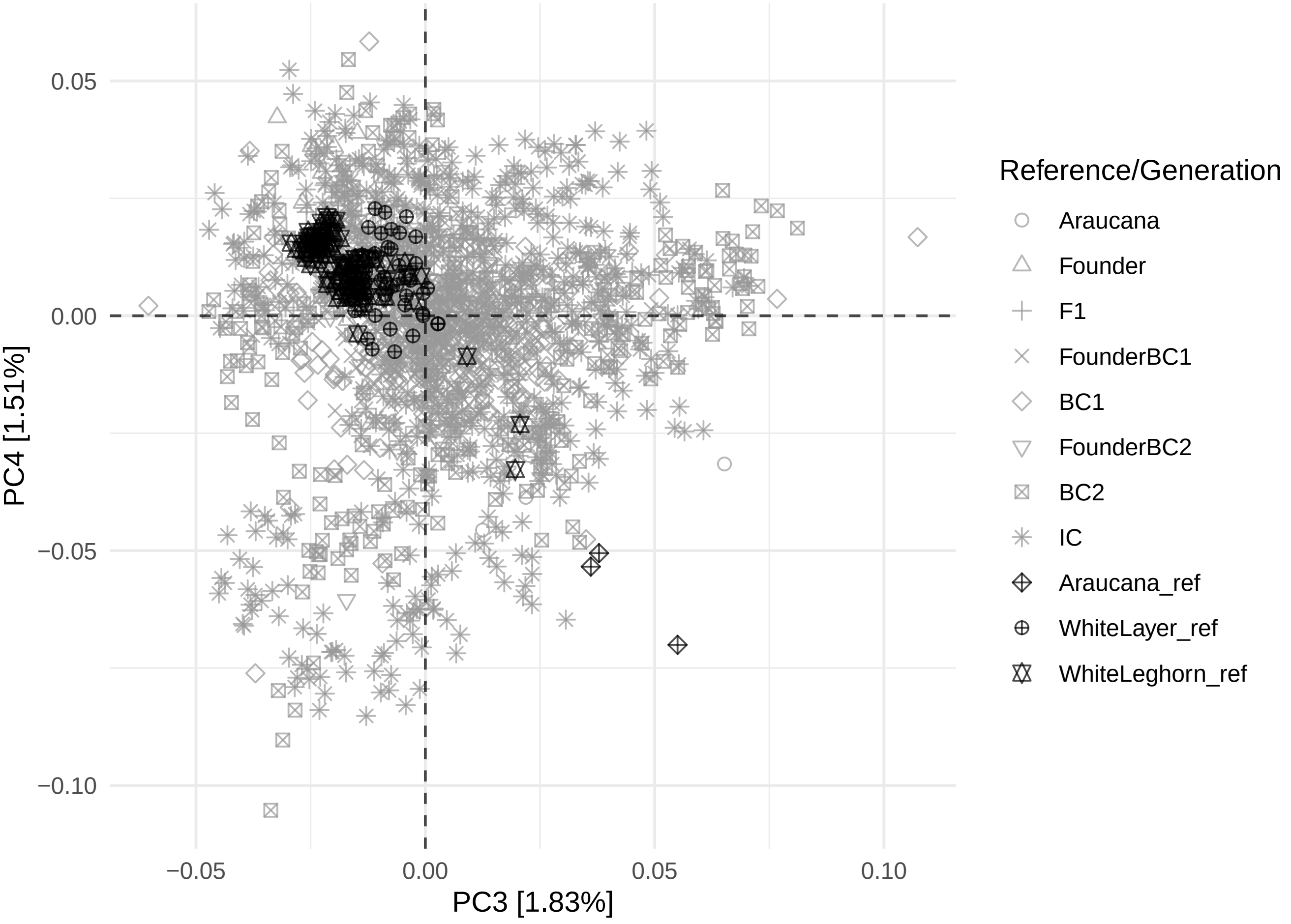

Reference/Generation
Araucana
Founder
F1
FounderBC1
BC1
FounderBC2
BC2
IC
Araucana_ref
WhiteLayer_ref
WhiteLeghorn_ref
PC4 [1.51%]
PC3 [1.83%]
0.05
0.00
−0.05
−0.10
−0.05
0.00
0.05
0.10
