# Supplementary material for: Bayesian fine-mapping pinpoints candidate genes and pleiotropic loci of production traits from a chicken backcrossing scheme
Source: BMC Genomics. 2026 Jun 16;27:548. doi: 10.1186/s12864-026-13069-z (PMC13270760; doi:10.1186/s12864-026-13069-z)
Supplement: Supplementary file 1 — Supplementary Material 1. Supplementary Figures 1: Combination of Manhattan plots of array and imputed GWAS across traits and groups. Supplementary Figures 2: Combination of Manhattan plots of array and imputed GWAS among groups, including BW32 as a covariate for EW (EW30, EW40, EW50, EW70) and vice versa. Supplementary Figures 3: Principal component analysis (PCA) of reference and target population used for imputation. Supplementary Figures 4: Visualization of phenotypic distribution between generations. Supplementary Data 1: Phenotypic records and generation of individuals. Supplementary Data 2: Result1 – Imputation accuracy across MAF bins (0, 0.01, 0.05, 0.1, 0.3, 0.5) accessed using five-fold cross-validation with two repetitions, including IQS, mean concordance, mean Pearson's correlation, minimum Pearson's correlation, and DR2 for each repetition and fold. Result2 – Mean genomic inflation factors for each trait and sample group combination of array and imputed GWAS. Result3 – Comparison of lead SNP selection on varying QTL windows of selected traits between BFMAP-FS and FINEMAP-sss. Result4 – QTLs of 17 traits for fine-mapping and the number of variants within the QTL region; from BFMAP: estimated QTL heritability and number of variants in the CS before and after signal filtration; from FINEMAP: number of variants with marginal PIP > 0, number of variants with marginal PIP > 0 and log10(BF) ≥ 2, posterior probability, log10(BF) quantifying evidence that there is an additional causal signal, and average absolute correlation between SNPs, for each model assuming the number of causal SNP is 1 or 2. Supplementary Data 3: BFMAP: Fine-mapping pinpointed genes before signal filtration with summed PCIP > 0.01, ordered by chromosomes, descending summed PCIP, and genes. FINEMAP: Fine-mapping pinpointed genes with summed PIP > 0.01, ordered by chromosomes, descending summed PIP, and genes. Supplementary Data 4: Enrichment analysis of BFMAP-FS results with combined trait [file 12864_2026_13069_MOESM1_ESM.zip › Supplementary files/Supplementary Figures 4.pdf]

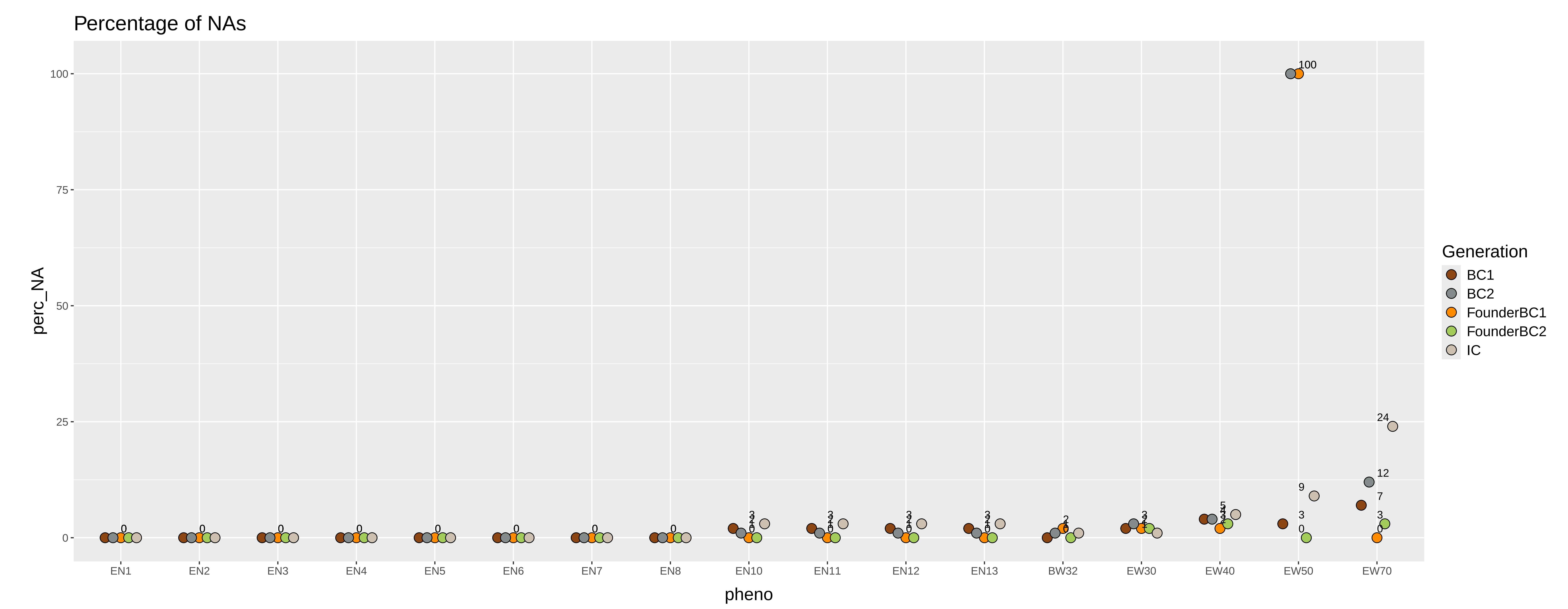
Percentage of NAs
perc_NA
100
75
50
25
0
pheno
EN1
EN2
EN3
EN4
EN5
EN6
EN7
EN8
EN10
EN11
EN12
EN13
BW32
EW30
EW40
EW50
EW70
Generation
BC1
BC2
FounderBC1
FounderBC2
IC
24
12
9
7

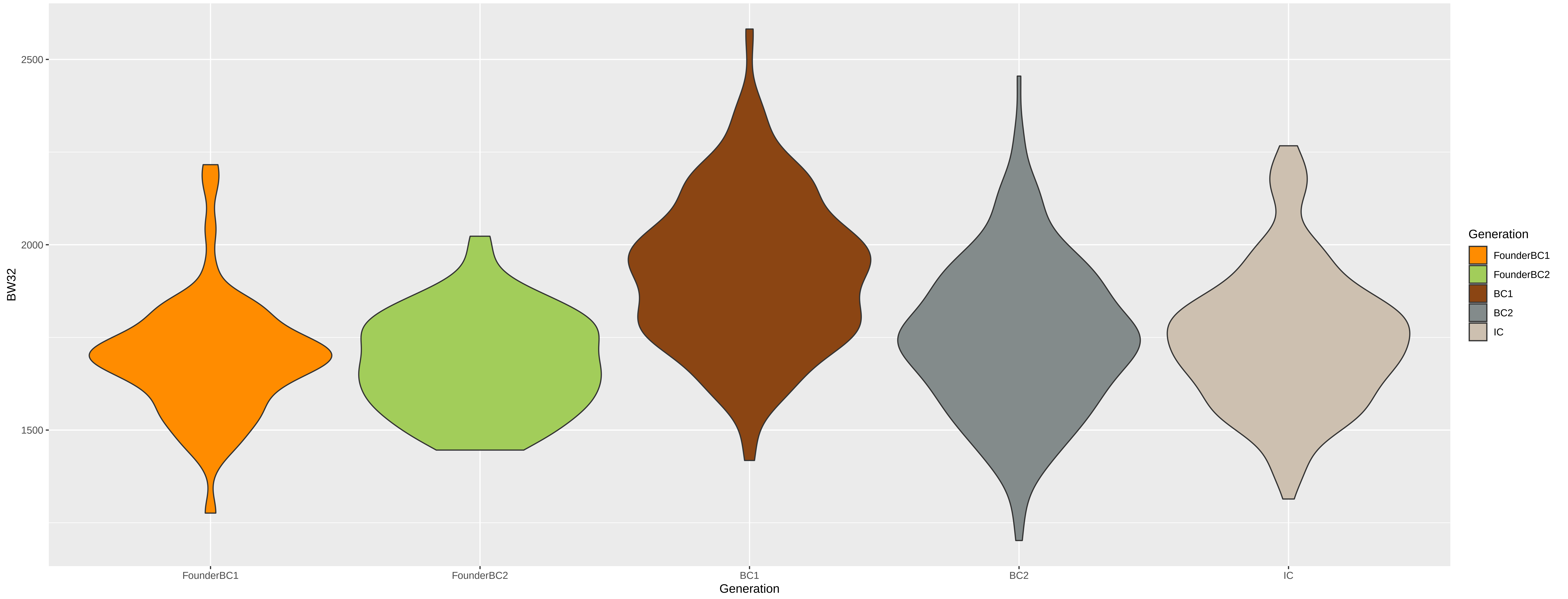

2500
2000
1500
BW32
FounderBC1
FounderBC2
BC1
BC2
IC
Generation
Generation
FounderBC1
FounderBC2
BC1
BC2
IC

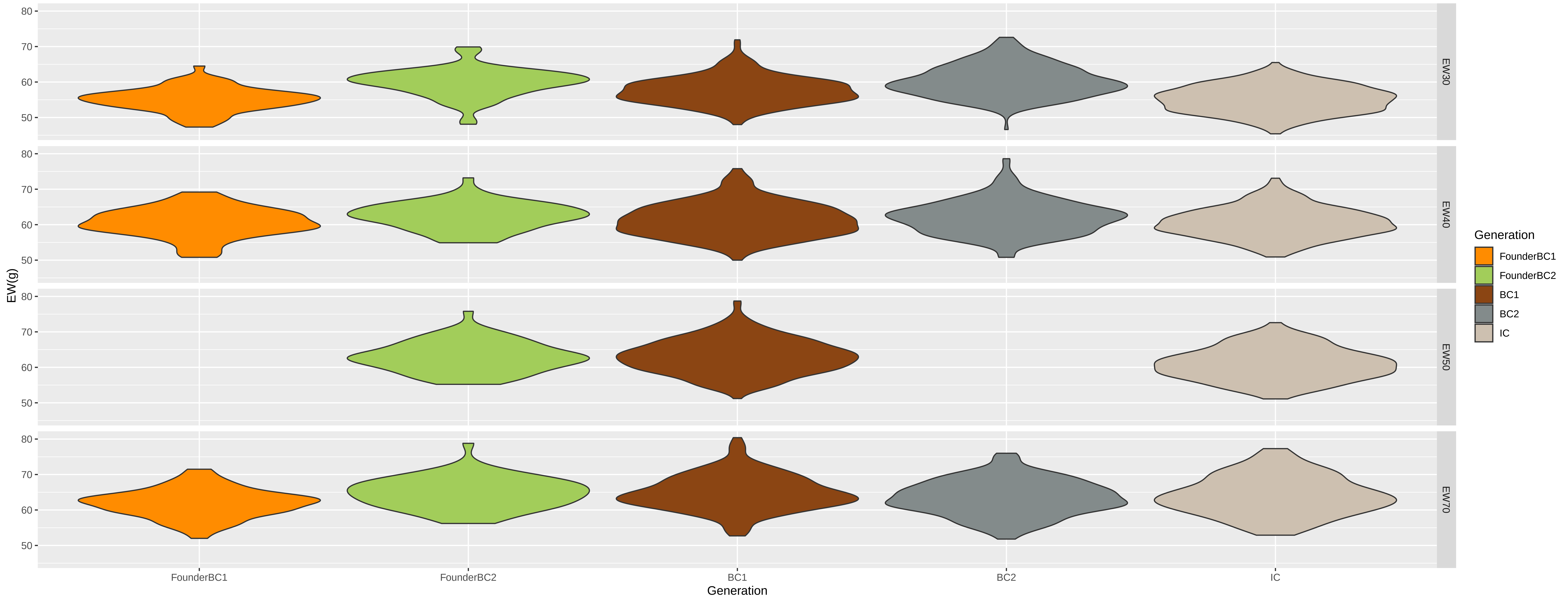

EW30
EW40
EW50
EW70
EW(g)
Generation
FounderBC1
FounderBC2
BC1
BC2
IC
80
70
60
50

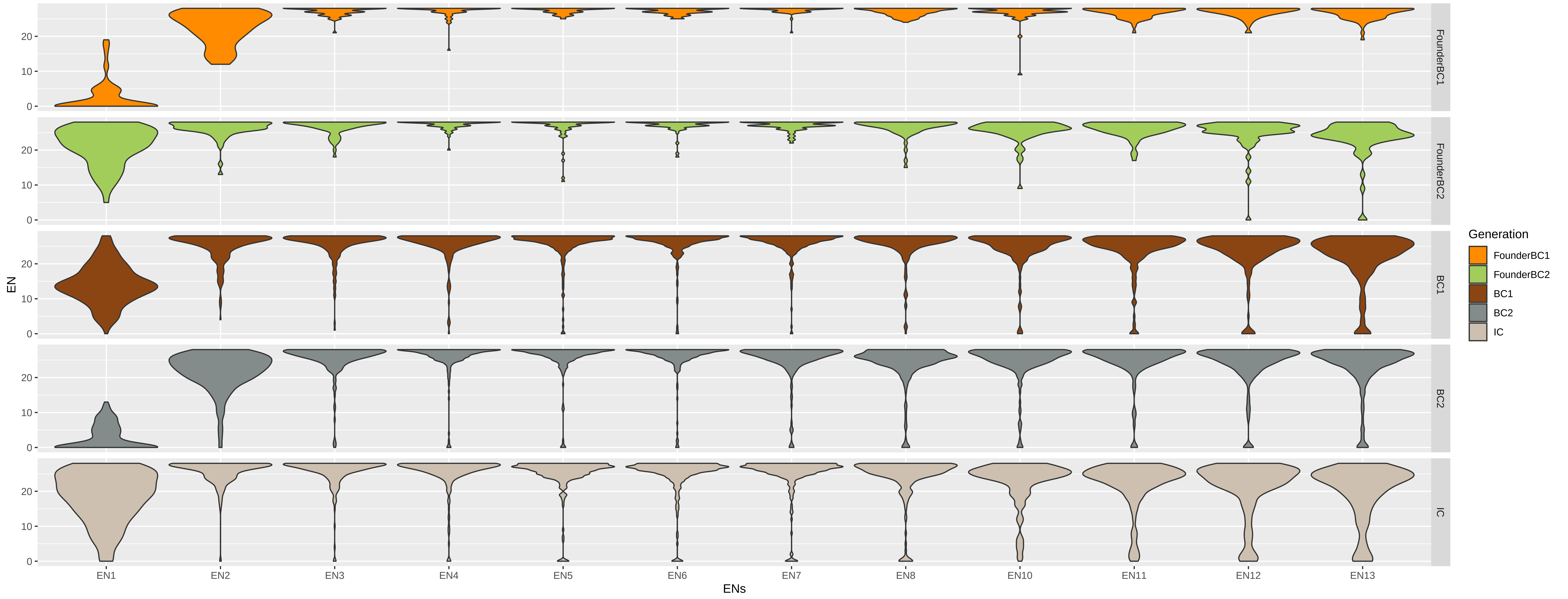

EN
ENs
EN1
EN2
EN3
EN4
EN5
EN6
EN7
EN8
EN10
EN11
EN12
EN13
FounderBC1
FounderBC2
BC1
BC2
IC
Generation
FounderBC1
FounderBC2
BC1
BC2
IC
0
10
20
